# Suppression of Spry1 reduces HIF1α-dependent glycolysis and impairs angiogenesis in BRAF-mutant cutaneous melanoma

**DOI:** 10.1186/s13046-025-03289-8

**Published:** 2025-02-14

**Authors:** Barbara Montico, Giorgio Giurato, Roberto Guerrieri, Francesca Colizzi, Annamaria Salvati, Giovanni Nassa, Jessica Lamberti, Domenico Memoli, Patrizia Sabatelli, Marina Comelli, Arianna Bellazzo, Albina Fejza, Lucrezia Camicia, Lorena Baboci, Michele Dal Bo, Alessia Covre, Tuula A. Nyman, Alessandro Weisz, Agostino Steffan, Michele Maio, Luca Sigalotti, Maurizio Mongiat, Eva Andreuzzi, Elisabetta Fratta

**Affiliations:** 1 Immunopathology and Cancer Biomarkers Unit, Centro di Riferimento Oncologico di Aviano (CRO), IRCCS, Aviano, Italy; 2 Department of Medicine, Surgery and Dentistry ‘Scuola Medica Salernitana’, Laboratory of Molecular Medicine and Genomics, University of Salerno, Baronissi, Italy; 3 Genome Research Center for Health - CRGS, 84081 Baronissi, SA Italy; 4 Division of Oncology, AOU ‘S. Giovanni Di Dio E Ruggi 14 d’Aragona’, Università Di Salerno, Molecular Pathology and Medical Genomics Program, Salerno, 84131 Italy; 5 CNR-Institute of Molecular Genetics “Luigi Luca Cavalli-Sforza”, Unit of Bologna, Bologna, Italy; 6 Department of Medicine, University of Udine, Udine, Italy; 7 Molecular Oncology Unit, Centro Di Riferimento Oncologico Di Aviano (CRO), IRCCS, Aviano, Italy; 8 UBT-Higher Education Institution, Street Rexhep Krasniqi Nr. 56, Prishtina, Kalabria 10000 Kosovo; 9 Experimental and Clinical Pharmacology Unit , Centro Di Riferimento Oncologico Di Aviano (CRO), IRCCS, Aviano, PN Italy; 10 University of Siena, Siena, Italy; 11 Department of Immunology, University of Oslo and Oslo University Hospital, Oslo, Norway; 12 Center for Immuno-Oncology, University Hospital of Siena, Siena, Italy; 13 Oncogenetics and Functional Oncogenomics Unit, Centro Di Riferimento Oncologico Di Aviano (CRO), IRCCS, Aviano, Italy; 14 Obstetrics and Gynecology, Institute for Maternal and Child Health - IRCCS “Burlo Garofolo”, Trieste, 34137 Italy

**Keywords:** Spry1, HIF1α, Cutaneous melanoma, BRAF, Mitochondria

## Abstract

**Background:**

About 50% of cutaneous melanoma (CM) harbors the activating BRAF^V600^ mutation which exerts most of the oncogenic effects through the MAPK signaling pathway. In the last years, a number of MAPK modulators have been identified, including Spry1. In this context, we have recently demonstrated that knockout of Spry1 (Spry1^KO^) in BRAF^V600^-mutant CM led to cell cycle arrest and apoptosis, repressed cell proliferation in vitro, and reduced tumor growth in vivo. Despite these findings, however, the precise molecular mechanism linking Spry1 to BRAF^V600^-mutant CM remains to be elucidated.

**Materials and methods:**

Immunoprecipitation coupled to mass spectrometry was employed to gain insight into Spry1 interactome. Spry1 gene was knocked-out using the CRISPR strategy in the BRAF-mutant cell lines. Transmission electron microscopy was used to assess the relationship between Spry1 expression and mitochondrial morphology. By using in vitro and in vivo models, the effects of Spry1^KO^ were investigated through RNA-sequencing, quantitative real-time PCR, Western blot, and immunofluorescence analyses. The Seahorse XF24 assay allowed real-time measurement of cellular metabolism in our model. Angiogenic potential was assessed through in vitro tube formation assays and in vivo CD31 staining.

**Results:**

Spry1 was mainly located in mitochondria in BRAF^V600^-mutant CM cells where it interacted with key molecules involved in mitochondrial homeostasis. Spry1 loss resulted in mitochondrial shape alterations and dysfunction, which associated with increased reactive oxygen species production. In agreement, we found that nuclear hypoxia-inducible factor-1 alpha (HIF1α) protein levels were reduced in Spry1^KO^ clones both in vitro and in vivo along with the expression of its glycolysis related genes. Accordingly, Ingenuity Pathway Analysis identified “HIF1α Signaling” as the most significant molecular and cellular function affected by Spry1 silencing, whereas the glycolytic function was significantly impaired in Spry1 depleted BRAF^V600^-mutant CM cells. In addition, our results indicated that the expression of the vascular endothelial growth factor A was down-regulated following Spry1^KO^, possibly as a result of mitochondrial dysfunction. Consistently, we observed a substantial impairment of angiogenesis, as assessed by the tube formation assay in vitro and the immunofluorescence staining of CD31 in vivo.

**Conclusions:**

Altogether, these findings identify Spry1 as a potential regulator of mitochondrial homeostasis, and uncover a previously unrecognized role for Spry1 in regulating nuclear HIF1α expression and angiogenesis in BRAF^V600^-mutant CM.

**Significance:**

Spry1^KO^ profoundly impacts on mitochondria homeostasis, while concomitantly impairing HIF1α-dependent glycolysis and reducing angiogenesis in BRAF-mutant CM cells, thus providing a potential therapeutic target to improve BRAF^V600^-mutant CM treatment.

**Supplementary Information:**

The online version contains supplementary material available at 10.1186/s13046-025-03289-8.

## Introduction

In recent years, mounting evidence demonstrates that the Spry family members regulate several biological pathways, deeply affecting physiological and pathological processes [1]. Several studies indicate that the pleiotropic activities of the Spry proteins can exert both tumor suppressive or tumor promoting functions, depending on the cellular context [2–15]. In particular, Spry1 expression was found to promote a malignant phenotype in different tumors, including rhabdomyosarcoma [6], glioma [12], acute myeloid leukemia [13], breast [16] and pancreatic cancers [14]. Along this line, we have recently reported that Spry1 expression was significantly elevated in metastatic cutaneous melanoma (CM) respect to the primary tumor. Furthermore, Spry1 was overexpressed in 15% of the BRAF^V600^-mutant CM [10]. Although the MAPK/ERK pathway has been initially considered to be the main target of Spry 1 [1], in our previous study, the knockout of Spry1 (Spry1^KO^) not only increased the phosphorylation of ERK1/2, but also strongly activated p38 in BRAF-mutant CM [10]. Besides its ability to modulate MAPK signaling pathways, Spry1^KO^ markedly induced cell cycle arrest and apoptosis, repressed cell proliferation in vitro, and impaired tumor growth in vivo, suggesting that Spry1 may display an oncogenic function in this tumor type. Importantly, loss of Spry1 resulted in an increase of reactive oxygen species (ROS), thus improving the sensitivity to the treatment with BRAF inhibitors [10]. Interestingly, results from a study on glioblastoma multiforme have suggested that Spry1 expression might be closely associated with tumor angiogenesis [12]. Despite these findings, however, the impact of Spry1 on the tumor microenvironment (TME) and angiogenesis in CM has not been explored yet.

The hypoxia-inducible factor-1 alpha (HIF1α) is one of the major drivers of angiogenesis in tumor cells. HIF1α is often up-regulated in metastatic CM, and BRAF-mutation is known to increase its expression [17]. Aberrant HIF1α activation has been associated with the more aggressive characteristics in CM [18, 19], where it acts as a mediator of a proliferative to invasive phenotypic switch [20]. In particular, HIF1α was shown to promote tumor-associated vascular remodeling by up-regulating the expression of target genes involved in angiogenesis, including VEGFA and matrix metalloproteinases (MMP) [21]. In addition, HIF1α regulates metabolic reprogramming in cancer cells. For instance, HIF1α activates the expression of genes encoding glycolytic enzymes, such as pyruvate dehydrogenase kinase 1 (PDK1), which usually phosphorylates and inhibits pyruvate dehydrogenase 1 to promote a glycolytic phenotype [22]. Consistently, several studies suggested so far that HIF1α and related genes could be promising targets for CM therapy [23]. HIF1α expression is not only induced in response to limited oxygen availability, but it is also regulated through different signaling pathways [23]. Interestingly, normoxic expression of HIF1α has been found in several cancer types, including CM, which constitutively harbors high levels of HIF1α [24–26].

Stemming from the above reported notions, here we aimed to investigate in more detail the specific requirement of Spry1 for BRAF^V600^-mutant CM development. Herein, for the first time, we report that Spry1 is mainly localized in mitochondria, where it contributes to mitochondrial homeostasis which plays a pivotal role in angiogenesis [27] and HIF1α stabilization [28]. Consistently, using different approaches, we also show that Spry1 loss compromises angiogenesis and results in a reduced HIF1α nuclear expression which, in turn, impairs the glycolytic metabolism. Considering that Spry1 is highly expressed in BRAF^V600^-mutant CM [10], these results disclose new insights on the molecular pathways regulating CM development.

## Results

### Spry1 localizes in the mitochondria and influences its homeostasis in BRAF-mutant CM

**Fig. 1:**
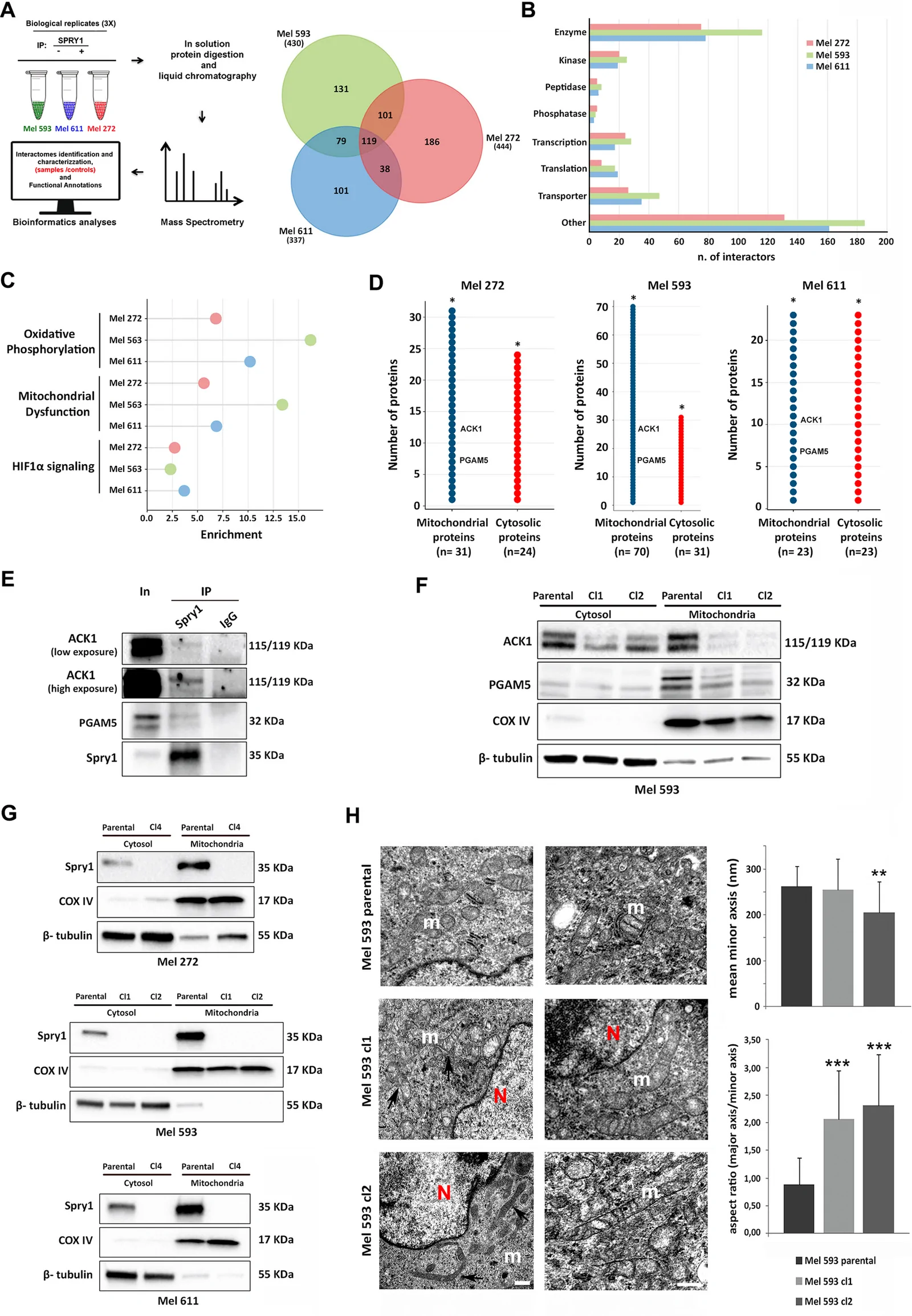
Spry1^KO^ is found associated to proteins involved in key mitochondrial functions in BRAF-mutant CM. **A** Summary of the experimental workflow applied to generate the protein datasets and venn diagram representing the Spry1-interacting proteins found enriched in Mel 272, Mel 593, and Mel 611 cells. **B** Classification of Spry1 molecular partners. **C** Lollipop chart showing statistically significant canonical pathways where the Spry1 interactomes are involved. The length of the lollipop indicates the enrichment value, the dot size corresponds to the number of proteins in each pathway. **D** Graph showing mitochondrial and cytosolic number of proteins in each of the Spry1 interactomes analysed; (* *P* ≤ 0.01, hypergeometric test). **E** IP-Western blot showing Spry1-ACK1 and Spry1-PGAM5 interaction. IgG was used as negative control. **F** Western blot analysis of ACK1, PGAM5 and Spry1 protein levels in cytosol and mitochondria in Mel 593 parental cells and Spry1^KO^ clones. COX IV and β-tubulin were used as protein loading control and as mitochondrial and cytosol markers, respectively. **G** Western blot analysis of Spry1 protein levels in cytosol and mitochondria in Mel 272, Mel 593, Mel 611 parental cells and Spry1^KO^ clones. COX IV and β-tubulin were used as protein loading control and as mitochondrial and cytosol markers, respectively. **H** TEM images of Mel 593 parental cells and their relative Spry1^KO^ clones. The arrows indicate m, mitochondrion; N, nucleus

We have previously demonstrated that Spry1 plays an oncogenic function in BRAF-mutant CM [10], however, how this is achieved is still poorly defined. To shed light on the molecular processes in which Spry1 takes part, interaction proteomics was performed in a panel of BRAF-mutant/NRAS wild-type (wt) CM cells, as outlined in Fig. 1A. To this aim, immunoprecipitation (IP) experiments were performed using an anti-Spry1 antibody or nonspecific IgGs as a control, and samples were subsequently subjected to proteomic analyses using liquid chromatography-tandem mass spectrometry (LC–MS/MS). Three independent biological replicates for each experimental condition were analyzed, in parallel. This resulted in the identification of 444, 430 and 337 Spry1 interacting partners within Mel 272, Mel 593 and Mel 611, respectively (Fig. 1A, Table S1A-C). Notably, 119 proteins were found to be commonly associated with Spry1 in all three cell lines. In each set of interacting proteins, enzymes, transcription regulators and transporters emerged as predominant categories (Fig. 1B). Functional enrichment analyses, performed by Ingenuity Pathway Analysis (IPA), on Spry1 interactomes in Mel 272, Mel 593 and Mel 611 cells highlighted the impact of Spry1 partners on mitochondrial dysfunction, oxidative phosphorylation (OXPHOS) and HIF1α signaling pathway (Fig. 1C). Consistently with the above-mentioned findings, for each set of Spry1 interactors, an enrichment of mitochondrial proteins was observed in Mel 272, Mel 593 and Mel 611 (Fig. 1D), including the non-receptor tyrosine kinase activated CDC42 kinase 1 (ACK1, also known as TNK2) and the mitochondrial serine/threonine protein phosphatase PGAM family member 5 (PGAM5) (Table S1A-C). Of note, the aberrant activity of these proteins might trigger mitochondrial dysfunction [29–34]. The interaction between ACK1, PGAM5 and Spry1 was next confirmed by independent co-IP experiments in Mel 593 cells, further validating our proteomics data (Fig. 1E). Importantly, the mitochondrial expression of ACK1 and PGAM5 were reduced following Spry1 loss (Fig. 1F). The direct interaction with mitochondrial proteins prompted us to further investigate the expression of Spry1 in this essential cellular compartment. To this end, we carried out Western blot analyses of mitochondrial fractions from parental and Spry1^KO^ CM cells, both exploiting clones that we have previously generated from Mel 272 and Mel 611 cell lines [10], and clones generated from the additional Mel 593 CM cell line (Fig. S1A). The latter had a phenotype that closely resembled the one observed in Mel 272 and Mel 611 clones following Spry1^KO^ [10] (Fig. S1B). Interestingly, results from the Western blot analyses indicated that Spry1 was abundantly localized in mitochondria (Fig. 1G), thus suggesting that Spry1 could play an important role in mitochondria homeostasis.

Since mitochondria functions are paralleled by their morphological features, we assessed the relationship between Spry1 expression and mitochondrial morphology. Transmission electron microscopy (TEM) was used to analyze the mitochondria shape in Mel 593 cells and the relative Spry1^KO^ clones. As observed in Fig. 1H, Mel 593 parental cells exhibited small and rounded-shaped mitochondria, whereas Spry1 silencing was associated with a significant elongation of the mitochondria, as assessed by the increase of the mitochondrial length (aspect ratio). Taken together, our results demonstrated that Spry1 plays a key role in maintaining the proper shape of mitochondria and the expression of important molecules regulating mitochondrial homeostasis in BRAF-mutant CM.

### Spry1 depletion associates with impaired cell metabolism and mitochondrial dysfunction in BRAF-mutant CM

**Fig. 2:**
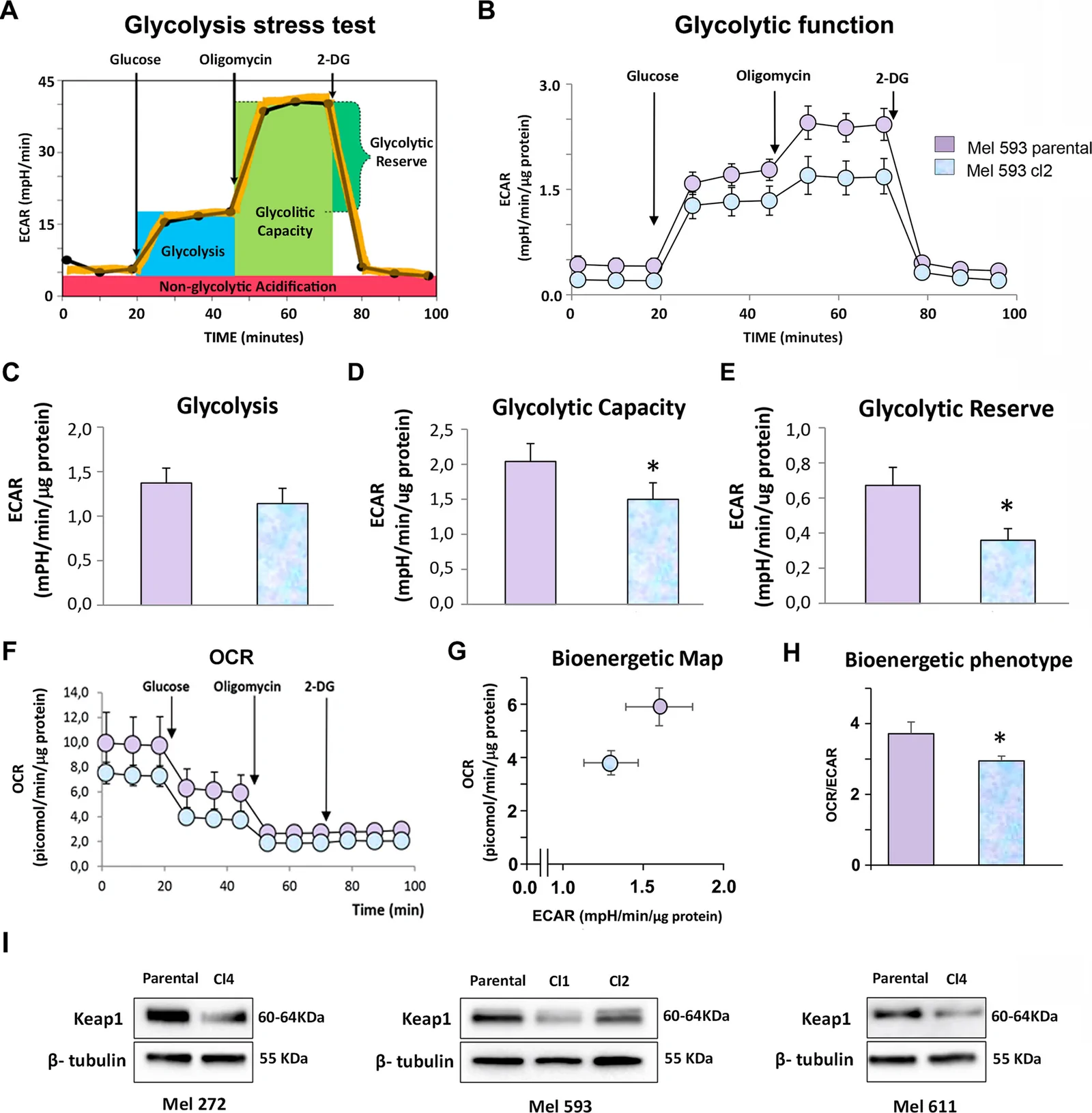
Spry1^KO^ impacts on metabolism and elevates the oxidative stress in BRAF-mutant CM. **A** ECAR was recorded over time in live cells with the Seahorse XFe24 analyser, as depicted in this scheme. The initial administration of a saturating concentration of glucose activates the glycolytic pathway. Protons induce a rapid increase in ECAR. The following inhibition of mitochondrial ATP synthesis with oligomycin shifts the energy production to glycolysis only and induces an additional increase in ECAR. Finally, inhibition of glycolysis with 2-DG causes a strong decrease in ECAR due to the non-glycolytic acidification of the medium. **B** Real-time ECAR was determined in Mel 593 parental cells and their relative Spry1^KO^ clones. One representative experiment is reported (from 3 different, independent experiments). Data were normalized with respect to protein content/well and reported as means ± SD, *n* = 5. **C-E** Values of basal gycolysis, maximal glycolytic capacity and glycolytic reserve respectively, of Mel 593 parental cells and their relative Spry1^KO^ clones. Bar plots are means ± SD, *n* = 5, from one experiment representative of three. Significance was evaluated with Student *t* test. **, *P* ≤ 0.01. **F** OCR was recorded simultaneously during ECAR detection, with the same injection protocol (glucose, oligomycin and 2-DG) in Mel 593 parental cells and their relative Spry1^KO^ clones. One representative experiment is reported (from 3 different, independent experiments). Data were normalized with respect to protein content/well and reported as means ± SD, *n* = 5. **G** Bioenergetic map (absolute OCR versus ECAR values) of Mel 593 parental cells and their relative Spry1^KO^ clones in the presence of glucose as substrate. Means ± SD, *n* = 5, for one experiment representative of three. **H** Bioenergetic phenotype (OCR/ECAR ratio) of Mel 593 parental cells and their relative Spry1^KO^ clones. This ratio is a qualitative measurement of the relative utilization of mitochondrial (oxidative) versus glycolytic pathway for energy production. Bar plots are means ± SD, *n* = 5, from one experiment representative of three. Significance was evaluated with Student *t* test. **, *P* ≤ 0.01. **I** Western blot analysis of Keap1 in Mel 272, Mel 593, and Mel 611 parental and respective Spry1^KO^ clones. β-tubulin was used as protein loading control

The shape of mitochondria can dynamically change in conjunction with metabolic disorders [35, 36], and the simultaneous upregulation of both glycolysis and OXPHOS has been shown to be vital for CM progression [37]. To determine whether the knockout of Spry1 affected glucose metabolism in our model, the extracellular acidification rate (ECAR), an indicator of the glycolytic function, was analyzed via the Glycolysis Stress Test by Seahorse XF24 (Fig. 2A and B) in Mel 593 parental and in Spry1^KO^ cells (clone 2). The basal glycolytic activity was impaired but not significantly, while the glycolytic capacity and the glycolytic reserve were notably reduced in Spry1 depleted CM cells (Fig. 2C-E). Next, to assess the relative contribution of glycolysis and OXPHOS to energy generation, in Mel 593 parental and in Spry1^KO^ cells we considered the oxygen consumption rate (OCR) (Fig. 2F) and the ECAR to evaluate the bioenergetic map (Fig. 2G) and the bioenergetic phenotype as OCR/ECAR ratio (Fig. 2H). Surprisingly, Mel 593 Spry1^KO^ cells exhibited a significantly impaired mitochondrial respiration and OCR/ECAR ratio, clearly indicating that mitochondrial bioenergetics was also impaired following Spry1 loss.

Mitochondrial dysfunction is usually accompanied by excessive ROS production, which can further damage mitochondria structure and metabolic function [38–40]. Interestingly, we previously found that ROS levels were significantly higher in Spry1^KO^ clones respect to the parental cell line [10]. Consistently, Mel 593 exhibited increased ROS production following Spry1 silencing (Fig. S2A). Furthermore, Keap1, which expression can be modulated through ROS-induced post-translational modifications [43], was down-regulated upon Spry1 deletion in Mel 272, Mel 593 and Mel 611 cell lines (Fig. 2I). Taken together, these results further indicate that mitochondrial dysfunction resulting from Spry1 loss promotes a metabolic reprogramming in BRAF-mutant CM.

### Spry1^KO^ associates with reduced HIF1 ɑ promoter activity and expression in BRAF-mutant CM

**Fig. 3:**
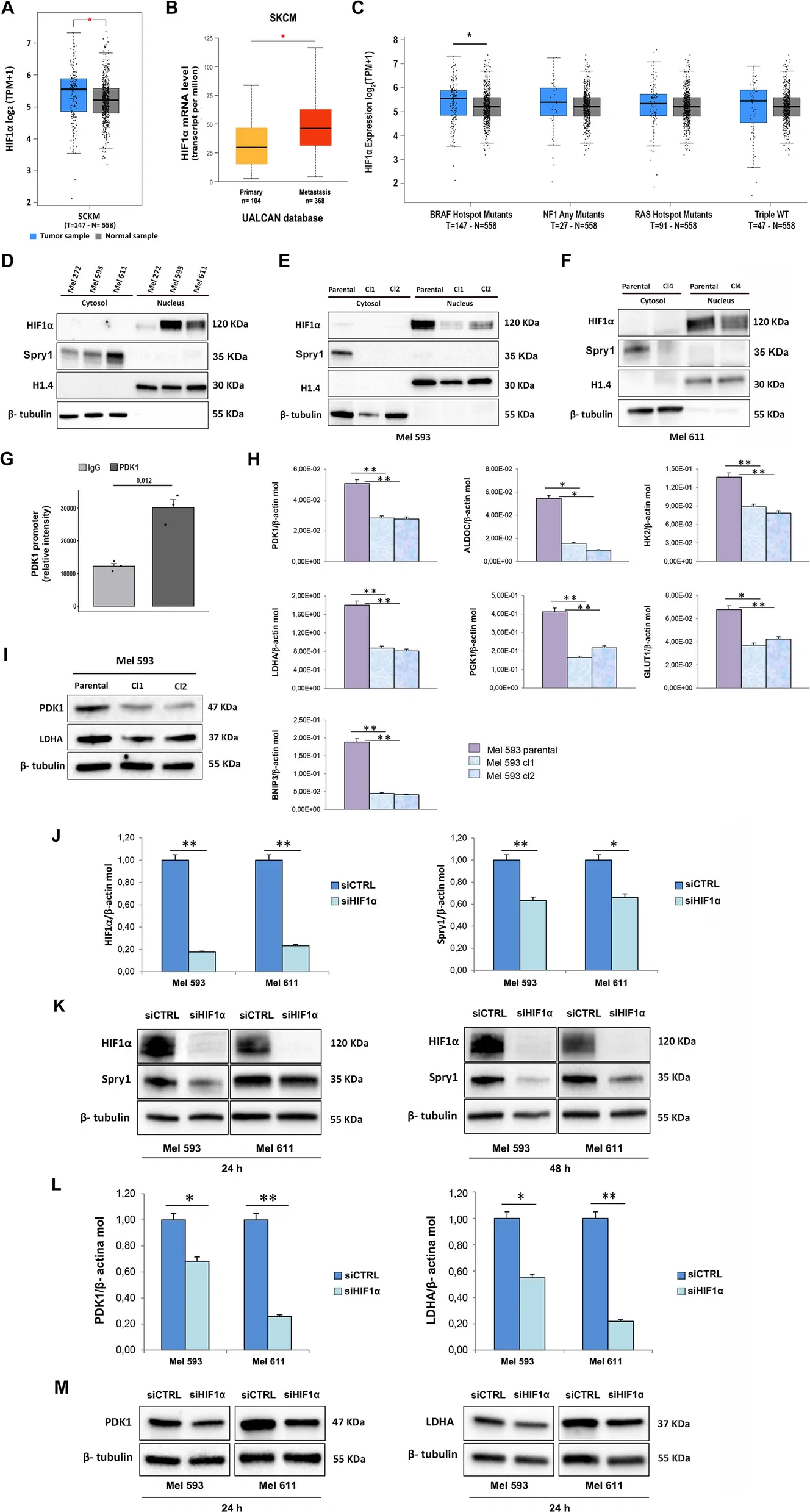
Spry1^KO^ reduces the expression and HIF1α effects on target gene transcription in BRAF-mutant CM. **A** Boxplot showing the expression of HIF1α gene in tumor and normal samples from SKCM-TCGA dataset obtained by GEPIA2. *, *P* ≤ 0.05. **B** Box plot showing the expression of HIF1α gene in primary and metastatic CM considering data taken from UALCAN Database. *, *P* ≤ 0.05. **C** Boxplot showing the expression of HIF1α gene in tumor and normal samples of SKCM-TCGA dataset, considering the four mutant subtypes. *, *P* ≤ 0.05. **D** Western blot analysis of HIF1α and Spry1 levels in the cytosol and nucleus of Mel 272, Mel 593 and Mel 611. Histone H1.4 (H1.4) and β-tubulin have been used as nuclear and cytosolic control molecules, respectively. **E** Western blot analysis of HIF1α and Spry1 levels in the cytosol and nucleus of Mel 593 parental and Spry1^KO^ clones 1 and 2. Histone H1.4 (H1.4) and β-tubulin have been used as nuclear and cytosolic control molecules, respectively. **F** Western blot analysis of HIF1α and Spry1 levels in the cytosol and nucleus of Mel 611 parental and Spry1^KO^ clone 4. Histone H1.4 (H1.4) and β-tubulin have been used as nuclear and cytosolic control molecules, respectively. **G** Validation of the ChIP-Seq results by PCR for the selected HIF1α binding sites within PDK1 gene in Mel 593 parental cells. **H** qRT-PCR analyses of ALDOC, BNIP3, GLUT1, HK2, LDHA, PDK1 and PGK1 expression in Mel 593 parental cells and relative Spry1^KO^ clones. Total RNA was extracted for reverse transcription, analyzed by qRT-PCR, and normalized to β-actin. Significance was evaluated with Student *t* test. *, *P* ≤ 0.5, **, *P* ≤ 0.01. **I** Western blot analysis of LDHA and PDK1 in Mel 593 parental cells and relative Spry1^KO^ clones. β-tubulin was used as a loading control. **J-M** Mel 593 and Mel 611 parental cells were transiently transfected with HIF1α-specific siRNA (siHIF1α) or nonspecific siRNA (siCTRL). The mRNA expression levels of HIF1α and Spry1 were measured 24 h after siRNA transfection. Total RNA was extracted for reverse transcription, analyzed by qRT-PCR, and normalized to β-actin. Significance was evaluated with Student *t* test. *, *P* ≤ 0.5, **, *P* ≤ 0.01 (**J**). Protein levels of HIF1α and Spry1 were assessed using Western blot 24 and 48 h after siRNA transfection. β-tubulin was used as a loading control (**K**). The mRNA expression levels of PDK1 and LDHA were measured 24 h after siRNA transfection. Total RNA was extracted for reverse transcription, analyzed by qRT-PCR, and normalized to β-actin. Significance was evaluated with Student *t* test. *, *P* ≤ 0.5, **, *P* ≤ 0.01 (**L**). Protein levels of PDK1 and LDHA were monitored using Western blot 24 h after siRNA transfection. β-tubulin was used as a loading control (**M**)

Alterations in mitochondria dynamics likely impair HIF1α stabilization and reduce the expression of its downstream targets, including those involved in glucose uptake and metabolism [28]. HIF1α is often up-regulated in CM respect to normal tissues (Fig. 3A), and in metastasis compared to primary tumor (Fig. 3B). Importantly, BRAF mutations are known to increase its expression in CM (Fig. 3C; ref. [17]). Prompted by the IPA enrichment analyses highlighting an involvement of Spry1 in the HIF1α pathway, we next performed Western blot analyses in BRAF-mutant Mel 272, Mel 593, and Mel 611 cells to further dissect the impact of Spry1 on HIF1α expression in CM cells. The results from these analyses revealed a heterogeneous expression of constitutive HIF1α in the nuclei, with Mel 272 cells expressing the lowest level (Fig. 3D). Subsequent analyses confirmed that Spry1 silencing was associated with reduced nuclear HIF1α levels in Mel 593 and in Mel 611 cells, particularly in Mel 593 Spry1^KO^ clones (Fig. 3E and F). Hence, RNA-seq analysis was performed using parental and Spry1^KO^ Mel 593 cells, and a total of 4654 differentially expressed transcripts (fold-change cut of |1.5|), were identified in Mel 593 Spry1^KO^ (Table S2).

To investigate, in detail, genes that were transcriptionally affected by HIF1α in our in vitro model, we performed a ChIP-Seq analysis for HIF1α in parental Mel 593 cells (Fig. S3A-C), which unveiled a total of 26,506 HIF1α binding sites (Table S3A). Annotation of these sites against the GeneHancer track identified a significant association with promoters and enhancers (*p* value = 0.001, Z-score = 18,28, # of permutation = 1000) (Table S3B), pointing out the likely functional impact of this transcription factor in controlling gene expression in our cellular model. To identify among HIF1α target genes the ones whose expression could be affected by Spry1^KO^, we integrated transcripts significantly deregulated in Mel 593 Spry1^KO^ clone 2 with those showing a peak in their transcriptional unit or in an enhancer element potentially able to regulate it (Table S3C). Notably, among the identified genes emerged PDK1, one of the master regulators of glycolysis. PCR analysis further validated the HIF1α binding sites identified on the PDK1 gene (Fig. 3G), whereas qPCR and Western blot confirmed its down-regulation in Spry1^KO^ Mel 593 cells (Fig. 3H and I). These data suggest that, in our model, Spry1^KO^ might impact on glucose metabolism in CM through regulation of PDK1 via HIF1α. In line with these observations, lactate dehydrogenase A (LDHA) expression, which usually increases in cancer cells opting for glycolytic metabolism, was significantly reduced following Spry1 loss along with the mRNA levels of other glycolysis-related genes, such as aldolase C (ALDOC), hexokinase II (HK2), phosphoglycerate kinase 1 (PGK1), and the glucose transporter protein type 1 (GLUT1), which facilitates the transport of glucose into cells (Fig. 3H and I). As shown in Fig. 3H, BCL2 Interacting Protein 3 (BNIP3), which has been recently described as an upstream regulator of the pro‐tumorigenic HIF‐1α glycolytic program in CM cells [42], was also significantly downregulated in Spry1^KO^ clones.

A study by Hicks et al. has demonstrated that Spry2 expression is regulated by HIF proteins [43], thus prompting us to investigate whether HIF1α silencing might alter Spry1 mRNA and protein levels. To examine whether the expression of Spry1 was HIF1α dependent, HIF1α was knocked down using small interfering RNA (siRNA) in both Mel 593 and Mel 611 cell lines. siRNA-mediated HIF1α silencing resulted in a significant decrease of the endogenous Spry1 mRNA and protein expression (Fig. 3J and K). However, the absence of HIF1α-binding sites within promoter and/or regulatory components of Spry1 gene in ChIP-seq data led us to hypothesize that Spry1 regulation by HIF1α is indirect (Table S3A). Notably, PDK1 and LDHA were significantly reduced 24 h after siRNA-mediated HIF1α knockdown (Fig. 3L and M), thus confirming that HIF1α might control glycolysis metabolism in our model.

### Spry1^KO^ influences the pro-angiogenic properties of BRAF-mutant CM

**Fig. 4:**
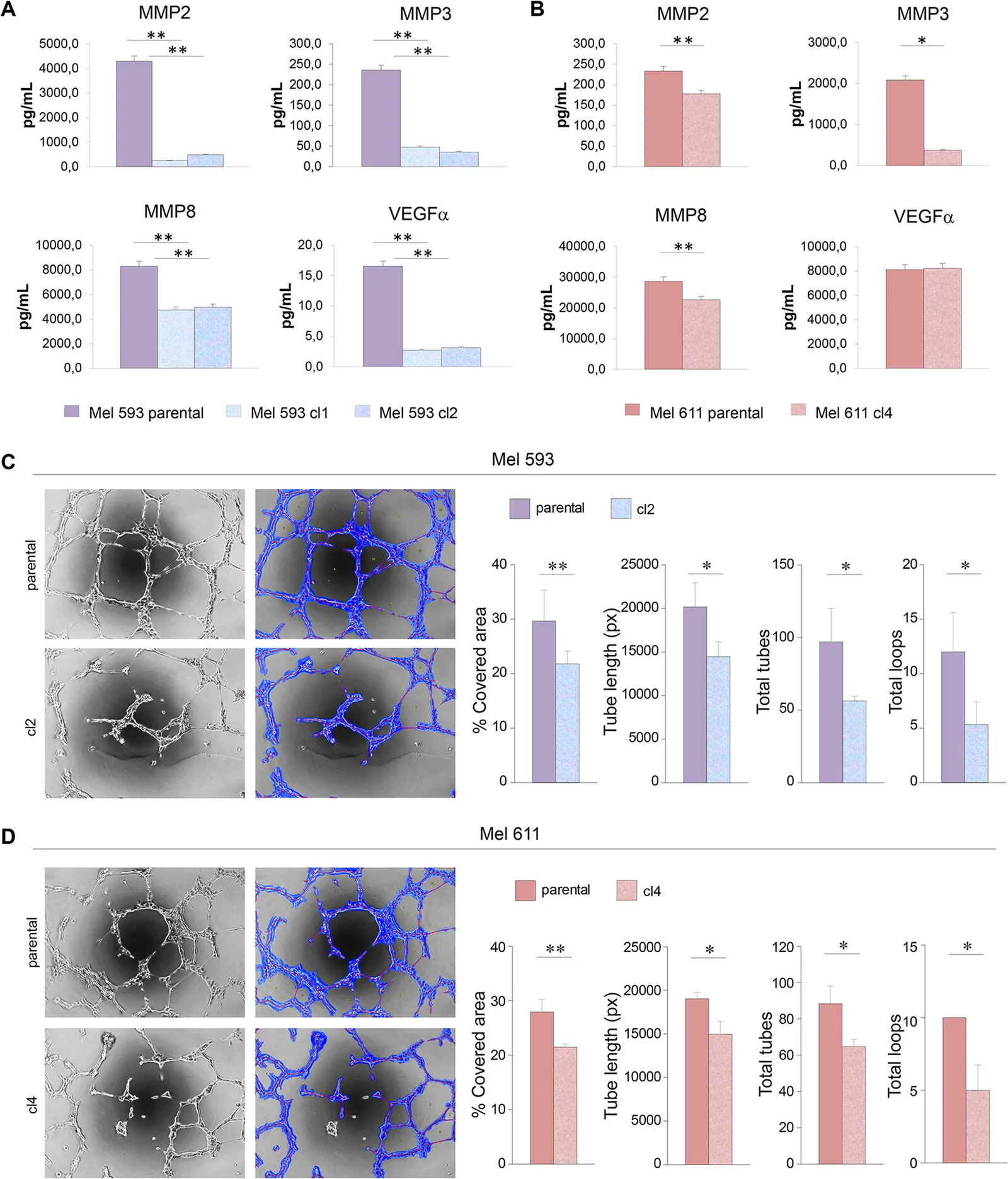
Spry1^KO^ affects tube formation in vitro. **A** and **B**, Mel 593 (**A**) and Mel 611 (**B**) parental and Spry1^KO^ cells were cultured for 48 h in RPMI 1640 complete medium supplemented with 2 mM L-Glutamine and 10% FBS. The media were then collected and MMP-2, MMP-3, MMP-8 and VEGF*ɑ* protein secretion was measured by a Luminex assay where experimental values were determined relative to a standard curve. Significance was evaluated with Student *t* test. *, *P* ≤ 0.05. **, *P* ≤ 0.01. **C**, left: representative images of the tubes formed by HUVECs placed on MATRIGEL® and challenged with conditioned media from Mel 593 parental and Spry1^KO^ cells. Right: quantification of the percentage covered area, the tube length, total tubes, and total loops; (px): pixels. **D**, left: representative images of the tubes formed by HUVECs placed on MATRIGEL® and challenged with conditioned media from Mel 611 parental and Spry1^KO^ Mel 611 cells. Right: quantification of the percentage covered area, tube length, total tubes, and total loops; (px): pixels

In addition to glycolytic enzymes, HIF1*ɑ* can directly or indirectly promote the transcription of angiogenic factors (i.e., VEGFA) [44]. Interestingly, although we found down-regulation of vascular endothelial growth factor A (VEGFA) expression and secretion following Spry1^KO^, this did not seem to directly involve HIF1* ɑ* in our model since ChIP-seq analysis did not identify any binding sites for this transcription factor within VEGFA promoter (Table S3A). Furthermore, differences in VEGFA secretion were not observed in Mel 611 upon Spry1^KO^, confirming that HIF1α did not regulate VEGFA expression in our model. Nevertheless, the secretion of other molecules implicated in the regulation of tumor angiogenesis in CM (e.g., MMP-2, MMP-3, MMP-8) [45] were significantly reduced in Spry1^KO^ Mel 593 clones (Fig. 4A and B).

To functionally verify the effect of Spry1 silencing on the angiogenic properties of Mel 593 and Mel 611 cells, we next carried out tube formation assays. To this aim, human umbilical vein endothelial cells (HUVEC) were challenged with conditioned media from parental and Spry1^KO^ cells and tube formation on matrigel was evaluated. Conditioned media from parental Mel 593 and Mel 611 promptly induced the formation of a net of tubes, despite with some differences (Fig. 4C and D). In fact, the most regular and organized tubes were observed when using conditioned media from Mel 593 cells. On the contrary, upon Spry1 silencing the conditioned media from Mel 593 and Mel 611 Spry1^KO^ clones failed to induce an optimal tube formation in terms of covered area, total tubes length, total tubes, and total loops (Fig. 4C and D). These evidences indicate that Spry1^KO^ significantly reduced the pro-angiogenic properties of CM cells.

### Spry1^KO^ alters HIF1α signaling pathway in BRAF-mutant CM in vivo

**Fig. 5:**
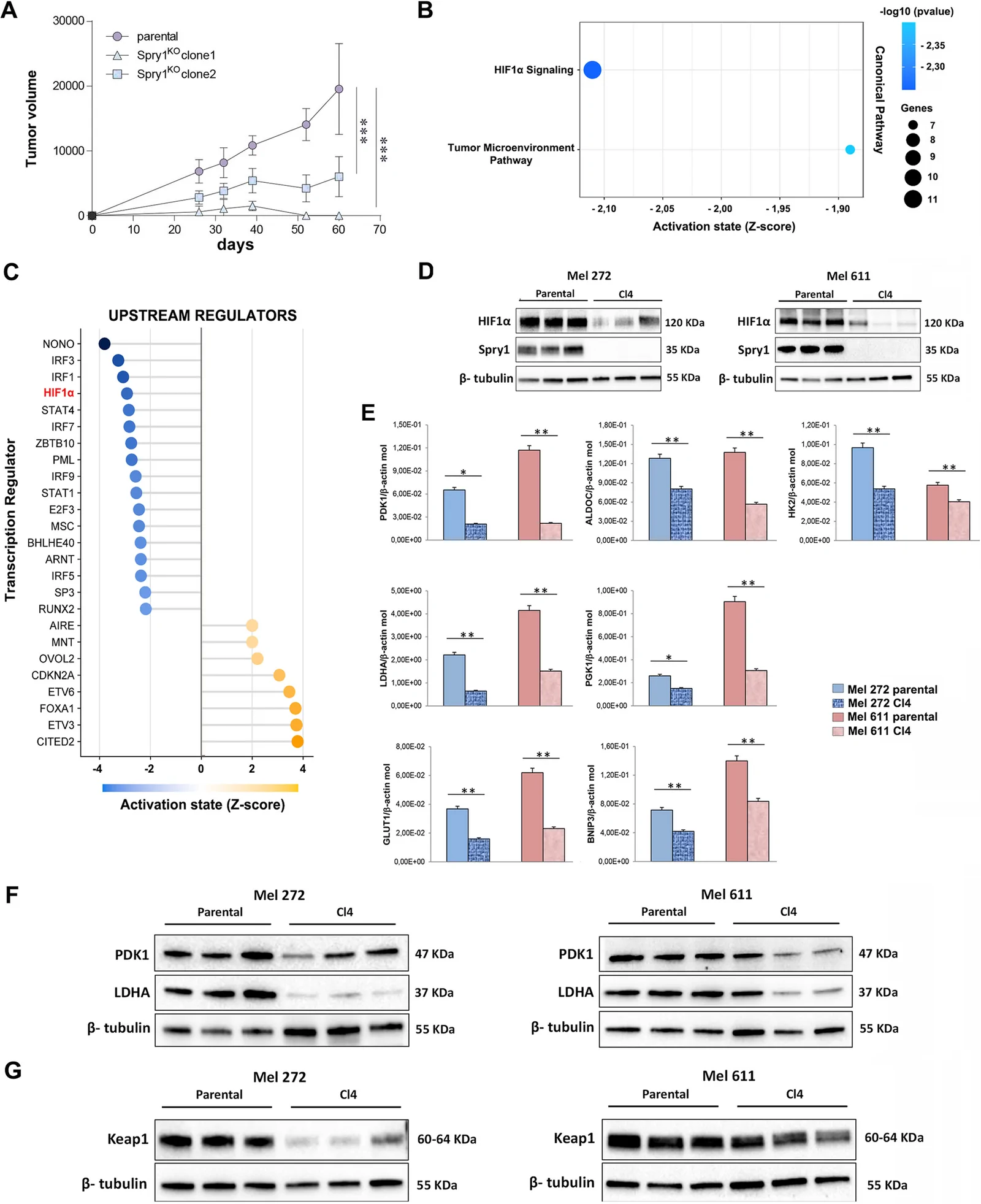
Spry1 loss influences HIF1α signaling in vivo. **A** Growth curve of Mel 593 parental and Spry1^KO^ clones 1 and 2 injected in athymic mice. **B** Lollipop chart showing the activation state (Z-score) of the HIF1α signaling and TME Pathway, depicting common differentially expressed transcripts in Spry1^KO^ Mel 272 and Mel 611 vs parental Mel 272 and Mel 611 tumor specimens. Dot size corresponds to the number of differentially expressed genes in each pathway, while the gradient colour indicates the associated log *p*-value. **C** Lollipop chart representing the activation state (Z-score) of the transcription regulators, resulted from the upstream regulator analysis performed considering the common differentially expressed transcripts between Spry1^KO^ vs parental Mel 272 and Mel 611tumor specimens. **D** Western blot analysis of HIF1α in tumor tissues obtained from parental and Mel 272 and Mel 611 Spry1^KO^ clones. β-tubulin was used as a loading control. **E** qRT-PCR analyses of ALDOC, BNIP3, GLUT1, HK2, LDHA, PDK1 and PGK1 expression in tumor tissues obtained from parental and Mel 272 and Mel 611 Spry1^KO^ clones. Total RNA was extracted for reverse transcription, analyzed by qRT-PCR, and normalized to β-actin. Significance was evaluated with Student *t* test. *, *P* ≤ 0.5, **, *P* ≤ 0.01. **F** and **G**, Western blot analysis of LDHA, PDK1 and Keap1 in the above mentioned tumor tissues. β-tubulin was used as a loading control

To verify whether Spry1 silencing could affect HIF1α signaling pathway in vivo, parental and Spry1^KO^ Mel 272, Mel 611 and Mel 593 cells were subcutaneously injected into the right flank of six-week-old female athymic nude mice. Wild type and Spry1^KO^ Mel 272 and Mel 611 cell growth was impaired as previously described [10], data not shown, similarly to what observed with Mel 593 cells, where, tumors arising from Spry1^KO^ clones were significantly smaller than those from the corresponding parental cells (Fig. 5A).

To gain insights on the molecular alterations occurring in TME upon loss of Spry1, RNA-seq analyses were performed. These analyses were only carried out in parental and Spry1^KO^ Mel 272 and Mel 611 tumors, since the extensive pigmentation of Mel 593 tumors (Fig. S4A) interfered with RNA analysis. With this approach we identified 571 transcripts differentially expressed between parental and Spry1^KO^ BRAF-mutant cells, among which 249 were down-regulated and 208 were up-regulated in both the cell lines (Table S4). Functional analysis of these common differentially expressed transcripts corroborated “HIF1α Signaling” as the most significantly inhibited canonical pathway (Fig. 5B). Consistently, upstream regulator analysis showed that HIF1α was ranked among the top ten inhibited transcription factors (Fig. 5C). In line with these results, protein analyses of tumor tissues revealed that the levels of HIF1α were strikingly reduced in Spry1^KO^ tumors (Fig. 5D), along with its glycolysis-related target genes (Fig. 5E and F, Fig. S4B). In addition to HIF1α, Keap1 levels were also affected (Fig. 5G, Fig. S4B), thus confirming that parental and Spry1^KO^ Mel 593 cells represented a suitable in vitro model, since they closely recapitulated the phenotype observed in vivo.

### Spry1^KO^ associates with a reduced vascularization of BRAF-mutant CM in vivo

**Fig. 6:**
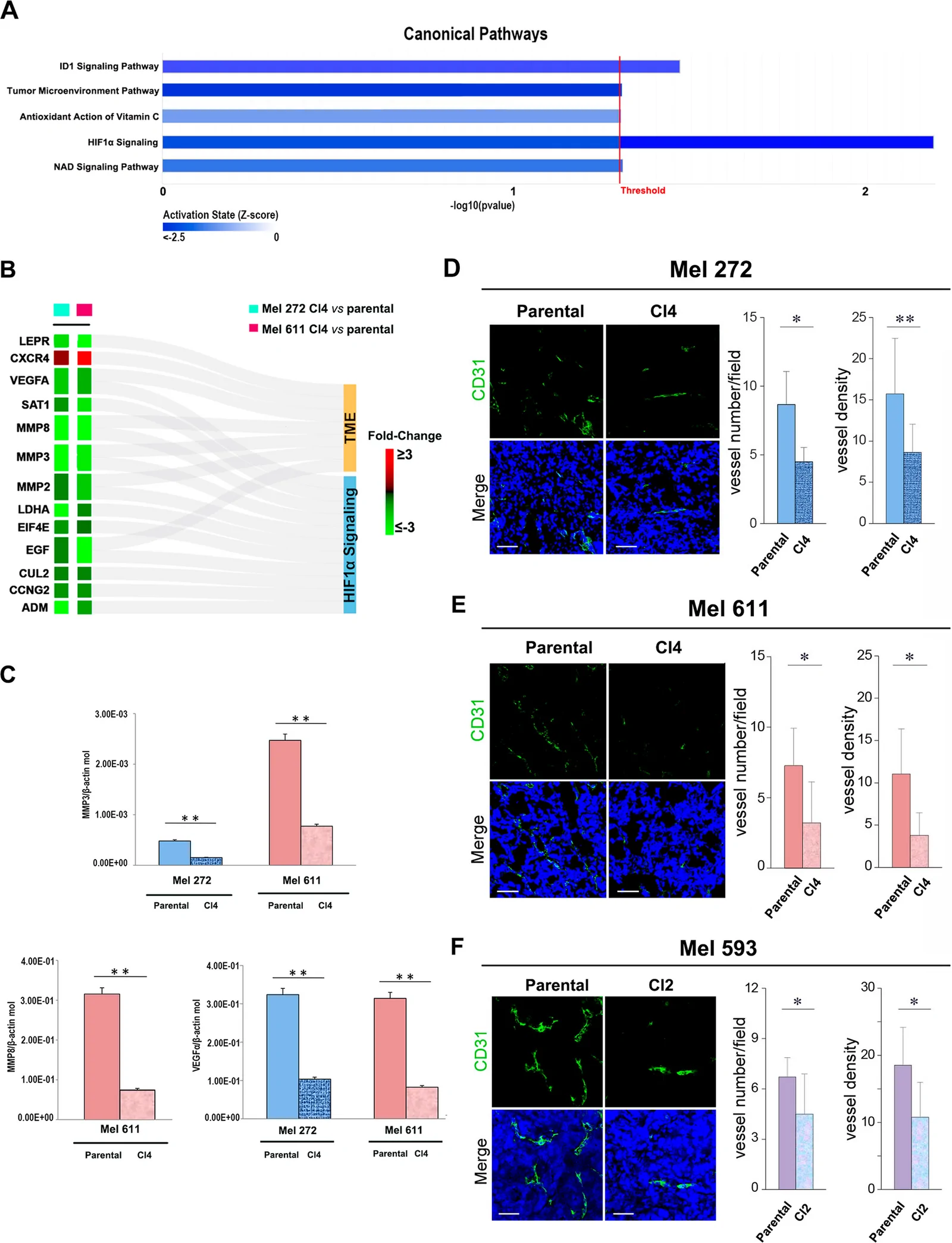
Spry1 silencing impacts on CM vascularization. **A** Graph depicting statistically significant canonical pathway enriched following Spry1^KO^.** B** Alluvial plot, showing the behaviour of commonly differentially expressed transcripts in Mel 272 and Mel 611 Spry1^KO^ clones vs parental specimens, belonging to HIF1α signaling and TME Pathway. **C** qRT-PCR analyses of MMP-3, MMP-8 and VEGFα expression in tumor tissues. Total RNA was extracted for reverse transcription, analyzed by qRT-PCR, and normalized to β-actin. Significance was evaluated with Student *t* test. **, *P* ≤ 0.01. **D** Representative immunofluorescence images of the endothelial cell marker CD31 (green) performed on xenograft tumors derived from Mel 272 parental and Spry1^KO^ clone 4. Blue: nuclei; scale bar = 100 µm. The graphs show the number of vessels per field and the vessel density calculated as the area covered by CD31 in respect to the area of total nuclei. **E** Representative immunofluorescence images of the endothelial cell marker CD31 (green) performed on xenograft tumors derived from Mel 611 parental and Spry1^KO^ clone 4. Blue: nuclei; scale bar = 100 µm. The graphs show the number of vessels per field and the vessel density calculated as the area covered by CD31 in respect to the area of total nuclei. **F** Representative immunofluorescence images of the endothelial cell marker CD31 (green) performed on xenograft tumors derived from Mel 593 parental and Spry1^KO^ clone 2. Blue: nuclei; scale bar = 100 µm. The graphs show the number of vessels per field and the vessel density calculated as the area covered by CD31 in respect to the area of total nuclei

Besides “HIF1α Signaling”, TME was one the pathways most significantly affected by Spry1 silencing (Figs. 5B and 6A). As further confirmation of these findings, MMP-2 was down-regulated under these conditions, as previously demonstrated in Spry1^KO^ clones both in vitro and in vivo [10]. Indeed, along with MMP-3, MMP-8 and VEGFA, MMP-2 was among the affected molecules contributing to both pathways (Fig. 6B). Although MMP-8 was barely expressed in Mel 272 tumors (data not shown), quantitative real-time PCR analysis confirmed that the mRNA levels of all these genes were significantly decreased in Mel 272 and Mel 611 Spry1^KO^ clones (Fig. 6C).

In accordance with the molecular analyses, during the excision of Mel 272 and Mel 611 tumors, a less evident vascularization was macroscopically observed in the lesions arising from Spry1^KO^ cells (Fig. S5) [10]. This preliminary observation was confirmed when the tumor-associated vessels were analyzed by immunofluorescence. Indeed, in both Mel 272 and Mel 611 tumors, the vessel number and the vascular density were significantly reduced when Spry1 was not expressed (Fig. 6D and E). Similar results were obtained in tumors from Mel 593 (Fig. 6F). These analyses could not be performed in tumors from Mel 593 clone 1 cells, since they completely regressed (Fig. 5A).

These findings corroborate the previous evidence indicating that Spry 1 exerts oncogenic functions within BRAF-mutant CM cells, highlighting for the first time the effect of Spry1 loss on the mitochondrial and metabolic processes. In addition, they unravel an unexpected loop whereby Spry1 and HIF1α mutually reinforce their pro‐tumorigenic function, leading to increased angiogenesis and profoundly impacting the TME, a key feature in promoting BRAF-mutant CM development and progression.

## Discussion

In the last years, it has become increasingly clear that Spry1 can display tumor promoting or suppressive functions depending on the cancer type and/or microenvironmental features. In this study, we unveiled a previously uncharacterized role for Spry1 in metabolic rewiring in BRAF-mutant CM occurring, at least partially, through the modulation of HIF1α stability. In fact, for the first time, we demonstrated that Spry1^KO^ was associated with a significant reduction of HIF1α expression.

Although HIF1α is usually rapidly degraded under normoxic conditions, several studies have reported that HIF1α is constitutively and stably expressed in CM cells not only during hypoxia [24, 25], and this occurs particularly in BRAF-mutant CM [17, 26], where the activation of the MAPK/ERK pathway has been correlated with HIF1α expression [46]. In our model, ERK1/2 was hyperactivated upon Spry1 abrogation (Fig. S1B) [10], thus suggesting that the normoxic expression of HIF1α might not be directly regulated by ERK. It has been postulated that HIF1α might play a different role under normoxic or hypoxic conditions [47]. In particular, the activity of HIF1α under normoxia seems to be responsible for the development of glycolytic tumors. Consistently, CM cells carrying the BRAF^V600^ mutation usually display a metabolic phenotype characterized by high glycolytic activity [48], which might be partially caused by the ability of HIF1α to enhance glycolysis, even in the presence of oxygen [17, 25]. Under the normoxic context of the in vitro experimental settings, Spry1 silencing reduced the ability of HIF1α to regulate its target genes, including PDK1, which represents a central regulatory switch at the crossroads of glycolysis to oxidative phosphorylation. These findings implicate Spry1 as a potential regulator of glycolysis, and were further confirmed in vivo since tumors from Spry1^KO^ cells exhibited very low levels of HIF1α and reduced expression of a number of glycolytic markers, including PDK1. Along this line, in a recent study by Raynor et al., the uptake of a glucose analogue was reduced in Spry1/2-deficient CD8^+^ T cells [49], suggesting that Spry1/2 loss might impair glucose metabolism. Nevertheless, the molecular mechanism was not investigated. On the other hand, Hicks et al. observed that Spry2 co-localized with HIF1α, and this co-localization was markedly decreased following HIF1α silencing. The same authors speculated that Spry2 enhanced HIF1α ubiquitylation and degradation by bringing pVHL in proximity of HIF1α [43]. Based on our data, we exclude that Spry1 directly interacts and/or co-localizes with HIF1α, which mainly accumulated in the nucleus. In fact, one of the interesting and novel observations from our studies is the localization of Spry1 in the mitochondria.

Prior to this study, not much was known about the function of Spry1 in mitochondria, since only a study by Alakoski et al. has reported this localization, despite in a different cell type [50]. Similarly to our previously published results [10], the same group found that Spry1 regulated p38 phosphorylation in cardiomyocytes [50]. p38/MAPK signaling is activated by essentially all environmental stresses, including oxidative stress and ROS production [51]. The results obtained by the analyses of Mel 593 cells confirmed the activation of p38 following Spry1 loss (Fig. S1B), and strengthen Spry1 involvement in ROS formation [10]. Excessive ROS generation can impact on altered mitochondrial structure, morphology and function. Consistently, Spry1^KO^ cells exhibited more elongated mitochondria, but also a lower bioenergetic capacity. Based on these findings, we speculate that high Spry1 expression in CM not only promotes glycolysis but also affects mitochondrial homeostasis. Of interest, the metabolic rewiring observed in Spry1^KO^ cells can render the tumor microenvironment less immunosuppressive [52]. Considering that glycolysis has been associated with decreased immune-infiltration [53], and immune-evasion [54], the potential contribution of Spry1 on immune resistance mechanisms should be addressed in future studies.

Although the mitochondrial control over HIF1α is still debated, it is known that mitochondrial dysfunction can reduce HIF1α stabilization along with the expression of its downstream targets [28], and several mitochondria-derived molecules were shown to modulate HIF1α stability and activity, including ROS [55]. Taken together, these findings support the hypothesis that mitochondria impairment might lead to the observed HIF1α down-regulation. However, additional experiments are required to establish the precise molecular mechanism through which Spry1 deficiency impacts on mitochondrial function and HIF1α expression. More importantly, this study opens new avenues towards the understanding of how Spry1 regulates mitochondrial dynamics in CM. Among Spry1 interacting partners, the phosphatase PGAM5 has been associated with poor survival in CM [56], implying it might contribute to CM progression. PGAM5 has emerged as a potential regulator of mitochondrial morphology [29, 30, 32]; in particular, PGAM5 knockdown caused mitochondrial elongation [57] and resulted in more fused mitochondria [29]. These findings led us to hypothesize that the mitochondrial elongation we observed upon Spry1^KO^ might partially depend on PGAM5 down-regulation. Furthermore, PGAM5 controls ROS homeostasis since it is intimately bound to proteins involved in the response to oxidative stress, being an intermediate of the Keap1-Nrf2 signalling pathway [31, 33]. On the other hand, Keap1 can induce ROS-dependent cell death by recruiting PGAM5 [58]. Nevertheless, although Spry1 loss associates with reduced Keap1 protein levels, the data from the co-IP analyses excluded a possible PGAM5-Keap1 interaction in our cell model (data not shown). In a recent paper by Zhang et al., PGAM5 was found to be constitutively associated with BNIP3 [59], whose expression was significantly downregulated both in vitro and in vivo in our models. Intriguingly, in a study by Vara-Pérez et al., BNIP3 silencing reduced the HIF‐1α protein levels under normoxic conditions, and significantly diminished the expression of several glycolytic enzymes in CM cells [42]. Moreover, in a previous study by Maes et al., BNIP3 was shown to control the redox status in CM cells, most likely by removing the excess of ROS-producing mitochondria. Accordingly, BNIP3 silencing increased the mitochondrial mass and the baseline levels of ROS production [60]. Therefore, whether this novel functional link between Spry1 and PGAM5 might impact on the BNIP3/HIF1a axis in addition to ROS regulation is worthy of in-depth discussions and further studies. In addition to PGAM5, we provided experimental evidence that Spry1 physically interacted with ACK1, which has been recently demonstrated to phosphorylate the mitochondrial protein ATP5A1 [34], a key regulatory component of the mitochondrial respiratory chain. Hence, further studies are warranted to verify if ACK1 mediates ATP5A1 phosphorylation in CM cells.

The expression of the pro-angiogenic factor VEGFA has been correlated with the transition of CM lesions from a radial to a vertical growth phase, which is usually associated with CM progression [61]. In our in vivo model, tumors from Spry1^KO^ cells exhibited significantly reduced VEGFA mRNA levels and vascular density, and showed a slower growth rate respect to the tumors derived from parental cells [10]. On the other hand, although VEGFA secretion was not impaired in Mel 611 Spry1^KO^ cells, supernatants from Spry1 deficient cells were defective in inducing HUVEC tube formation, proving that Spry1 induces the secretion of several pro-angiogenic factors. Mitochondrial remodelling might reduce angiogenesis, thus influencing vessel formation [27, 62]. For instance, excessive ROS production resulting from mitochondrial dysfunction was shown to impair angiogenesis by inhibiting VEGFA expression and inducing endothelial alterations [27]. In this context, it has been demonstrated that excessive ROS production could inhibit BCL-2 expression and activity [63], which is known to enhance VEGF mediated angiogenesis [64, 65]. Hence, we can hypothesize that the reduced BCL-2 protein expression that we have observed following Spry1 loss (Fig. S1B) [10] might, at least in part, contribute to VEGFA down-regulation. Notably, MMPs overexpression has been associated with BCL-2 up-regulation, and increased microvascular density [45]. Furthermore, tumor angiogenesis frequently involves the cross-talk between VEGF and MMPs, which can also be transcriptionally regulated in a mutually coordinated manner [66]. Data generated from our studies clearly demonstrated that Spry1^KO^ led to the down-regulation of several MMPs, including MMP-2, MMP-3 and MMP-8, which have been recently explored as targets to hamper cancer angiogenesis and, in turn, tumor cell dissemination [45, 67–70]. Of interest, our results further indicate that MMPs might significantly contribute to angiogenesis and tumor dissemination through the induction of extracellular matrix remodelling [69]. This hypothesis is supported by the fact that Spry1^KO^ also induce a altered expression of extracellular matrix molecules, including collagens and different glycoproteins (Table S2), among which collagen IV and EMILIN-2 seem to be particularly relevant given their angiogenic and immunomodulatory functions [71].

## Conclusions

**Fig. 7:**
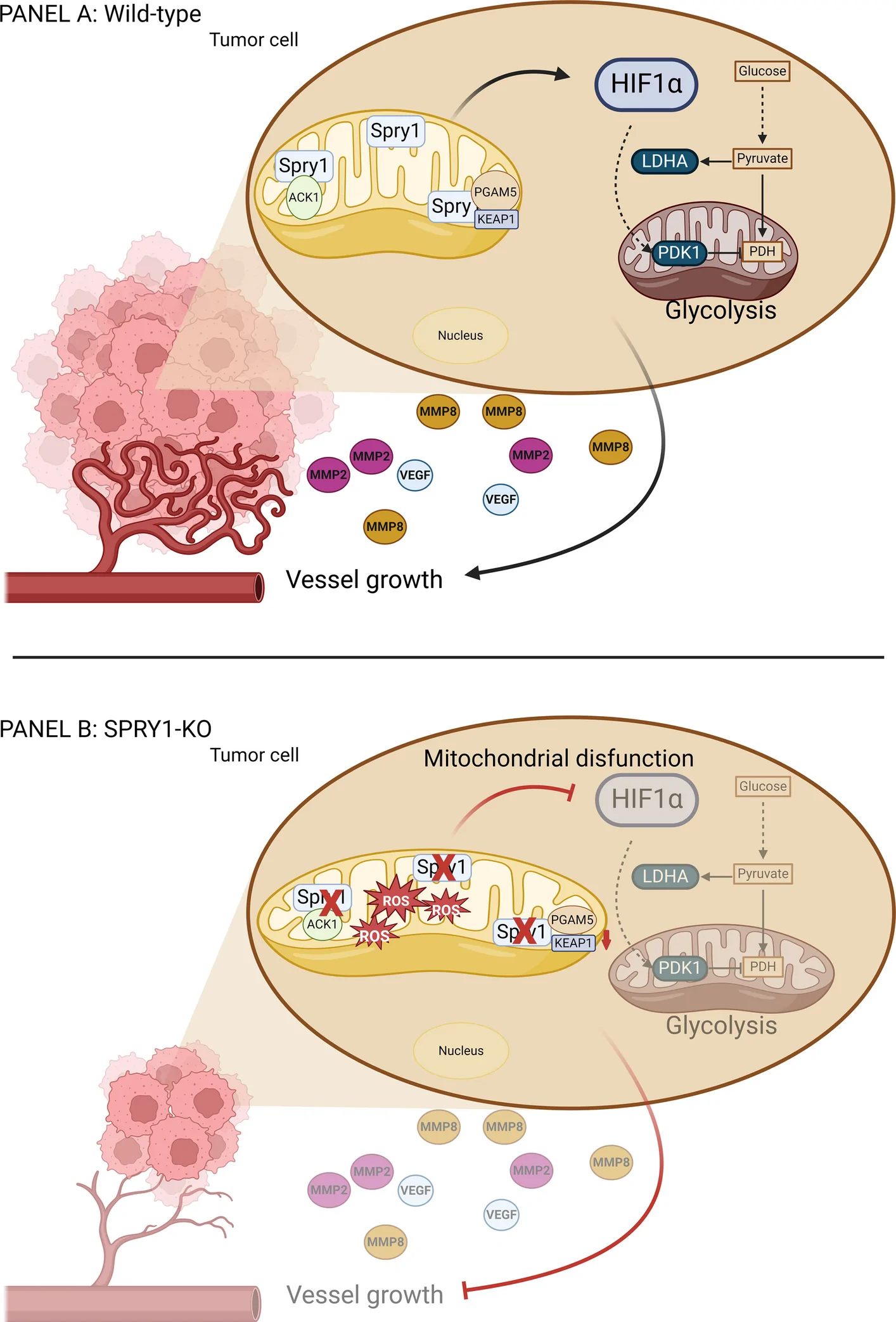
Spry1 maintains the angiogenic capacity of BRAF-mutant CM cells and contributes to HIF1α stabilization by controlling mitochondrial homeostasis. Consistently, Spry1 loss compromises angiogenesis and results in a reduced HIF1α nuclear expression which, in turn, impairs the glycolytic metabolism. Created in https://BioRender.com

In summary, we have unravelled an unexpected loop whereby Spry1 profoundly impacts on mitochondria homeostasis while concomitantly driving HIF1α-dependent glycolysis and inducing angiogenesis in BRAF-mutant CM cells (Fig. 7). Hence, this study further contributes to elucidate the potential role of Spry1 in CM pathogenesis and progression, thus providing the basis for new therapeutic options.

## Materials and methods

### Cells cultures and reagents

Cell cultures were established from biopsies of CM patients who were referred to the Centro di Riferimento Oncologico, IRCCS-National Cancer Institute, Aviano, Italy, as previously described [72]. Cells were cultured in RPMI-1640 Medium, with 10% fetal calf serum, 100 μg/mL streptomycin, and 100 IU/mL penicillin, and maintained at 37 °C under a humidified atmosphere containing 5% CO_2_. HUVEC were isolated from umbilical cord vein, as previously described [73], and cultured in ECM medium supplemented with 5% FBS and 1% Endothelial cell growth supplement, plus 100 I.U./ml of penicillin/streptomycin (ScienceCell). All cells were mycoplasma free. The study was approved by the Internal Review Board of the Centro di Riferimento Oncologico, IRCCS-National Cancer Institute, Aviano, Italy (IRB number 07–2017).

### In vivo tumor growth

Six-week-old female athymic nude mice from Envigo were subcutaneously injected in the flank with Mel 593 parental or Spry1^KO^ cells resuspended in 80 µl of PBS plus 20 µl of MATRIGEL® GFR (Franklin Lakes, NJ, USA) (1 × 10^6^ cells/animal). Animal health was monitored daily by observation and sentinel animal blood sample analysis. Tumor development was measured every 2–3 days for 69 days. At the end of the experiment, animals were sacrificed by CO_2_ overdose. Tumors were excised and material was divided for RNA and protein extraction, and included in OCT for subsequent immunofluorescence analyses. All in vivo studies were approved by the Italian Ministry of Health (no. 788/2015/PR).

### Immunofluorescence analyses

For immunofluorescence, serial cryostat 7 μM sections were obtained from OCT embedded tumor samples. Slices were collected on positively charged glass slides (Super-Frost Plus, VWR International S.r.l., Milan, Italy), air dried and fixed with 4% PFA for 5 min. After incubation with 0.5% Triton X-100 in PBS for 5 min at room temperature, the samples were blocked with 2% BSA in PBS for 1 h. Slides were next incubated overnight at 4 °C with primary antibody against CD31 (BD Bioscience). After washing, samples were incubated with secondary antibodies for 1 h at room temperature. Antibodies were added in the presence of TO-PRO™-3 (Invitrogen) to stain the nuclei. Following 3 washes with PBS, sections were mounted with Mowiol (Sigma Aldrich, Milan, Italy) supplemented with 2.5% 1,4 diazabicyclo(2,2,2)-octane (DABCO, Sigma Aldrich, Milan, Italy). Immunofluorescence images were acquired with a Leica TCS SP8 Confocal System (Wetzlar, Germany). Fluorescence intensity and quantification were evaluated by means of the Volocity software.

### Tube formation assay

To prepare the CM conditioned media, Mel 593 and Mel 611 confluent cells were cultured in ECM without serum, after 48 h the medium was collected, centrifuged to eliminate cell debris and freshly used. For tubuligenesis assay, 50 µL of MATRIGEL® GFR (Franklin Lakes, NJ, USA) were added to each well of a 96 well-plate and allowed to solidify for 30 min at 37 °C. HUVECs were serum starved for 4 h, treated with trypsin, harvested and resuspended in the conditioned media deriving from Mel 593 and Mel 611. HUVECs were then seeded onto the MATRIGEL® layer at a concentration of 1 × 10^4^ cells/well and cultures placed in the cell incubator (37 °C and 5% CO_2_) of a Leica Time Lapse AF6000LX workstation (Leica Microsystems GmbH,Wetzlar, Germany) interfaced with the Leica Application Suite software. Images were automatically taken every 15 min for 8 h and analyzed using online WimTube software (Wimasis Image Analysis, Ibidi, Onimagin Technologies SCA, Cordoba, Spain) to measure the covered area, the length of formed tubes, the number of total tubes and loops.

### Glycolysis stress test

Assays were performed using the Seahorse XFe24 analyzer (Seahorse, Agilent Technologies, Santa Clara, CA), according to the manufacturer’s instructions. Briefly, Mel 593 parental and Spry1^KO^ cells were seeded in 24 cell culture microplates (Seahorse, Agilent Technologies) at a concentration of 4 × 10^4^ cells/well in 500 μL of complete RPMI-1640 Medium and incubated for 24 h at 37 °C in 5% CO_2_ atmosphere. Before the experiments, the culture medium was removed from each well and replaced by 500 μl of Seahorse XF DMEM Medium, pH 7,4 (Seahorse, Agilent Technologies) pre-warmed at 37 °C and supplemented with 2 mM glutamine. Cells were incubated in a CO_2_-free incubator at 37 °C for 1 h, before the measurements. ECAR and OCR were measured following sequential additions of 10 mM glucose (to detect basal glycolysis), 1 μM oligomycin (to detect the maximal glycolytic capacity) and 50 mM 2-deoxy-D-glucose (2-DG) (to detect non-glycolytic acidification). Data were analysed by the Seahorse XF Glycolysis Stress Test Report Generator package. ECAR values were normalized to the protein content (μg) of each well obtained by the Bradford method.

### Transmission electron microscopy analyses

Mel 593 Spry1^KO^ clones and parental cells were grown to confluence, fixed with 2.5% glutaraldehyde in cacodylate buffer 0.1 M pH 7.4 for 2 h. To facilitate their handling as tissue fragments, cells (adherent or not) were collected by scraping and embedded in 4% gelatin in 0.1 M CB, as previously described [74]. Samples were post-fixed with 1% osmium tetroxide, dehydrated and embedded in Epon812 following standard procedures. Ultrathin sections were stained with uranyl acetate and lead citrate and observed with a Jeol JEM-1011 transmission electron microscope operated at 100 kV. For morphometrical analysis, 100 mitochondria for each sample were studied. Images were acquired with a CCD camera at the same magnification; aspect ratio (length-to-width ratio) as [(major axis)/(minor axis)] was calculated with the analysis software.

### Co-immunoprecipitation, mass spectrometry and data analysis

Mel 593 parental cells were grown in 15 cm culture dishes. Cells were collected and lysed in RIPA buffer supplemented with protease inhibitor cocktail and PMSF, following incubation on ice for 30 min. Samples were centrifuged at the highest speed for 15 min at 4 °C and protein lysate was collected. For Spry1 co-immunoprecipitation, anti-Spry1 or anti-IgG Isotype control were conjugated overnight at 4 °C with Dynabeads protein A (Thermofisher). Immunoprecipitation was performed by incubating conjugated beads/antibodies with protein lysates overnight at 4 °C. After incubation, samples were washed with IPP150 buffer (7.14 mM HEPES pH 7.5, 8.92% glycerol, 150 mM NaCl, 0.54 mM MgCl2, 0.07 mM EDTA pH 8 and protease inhibitors) and with Wash Buffer (50 mM Tris–HCl pH 7.6, 150 mM NaCl and protease inhibitors) and resuspended in 100 μl of 100 mM ammonium bicarbonate before tryptic digestion, LC–MS/MS and data analysis. MS analyses was performed on IP samples derived from three biological triplicates for Spry1 and IgG isotype control. Protein digestion was performed by on-beads digestion adding 0.5 μg trypsin (Promega) to each replicate followed by samples incubation at 37 °C overnight. Digested peptides were transferred to new tube, acidified and the peptides were desalted by homemade C18 stage tips. LC–MS/MS analysis was carried out using a nanoElute nanoflow ultrahigh pressure LC system (Bruker Daltonics, Bremen, Germany) coupled to the timsTOF fleX mass spectrometer (Bruker Daltonics), using a CaptiveSpray nanoelectrospray ion source (Bruker Daltonics). Raw files from LC–MS/MS analyses were submitted to MaxQuant 2.0.1.0 software for protein identification and label-free quantification. Parameters were set as follow: carbamidomethyl was set as a fixed modification and protein N-acetylation and methionine oxidation as variable modifications. First search error window of 20 ppm and mains search error of 6 ppm. Trypsin without proline restriction enzyme option was used, with two allowed miscleavages. Minimal unique peptides were set to one, and FDR allowed was 0.01 (1%) for peptide and protein identification. The Uniprot human database was used. Generation of reversed sequences was selected to assign FDR rate. Spry1 molecular interactors were obtained comparing each dataset to the control (IgG). We considered as Spry1 partners those molecules identified in at least 2 out of 3 sample biological replicates and absent in the controls or those supported by statistical analysis performed as follows: a permutation (10,000)-based T-test applied to MaxQuant protein ‘Intensities’ values (FDR ≤ 0.05) between samples (Spry1) and controls (IgG) and a fold change cutoff ≥ I quartile.

### Statistical analysis

At least three independent experiments were taken into consideration for statistical analyses and the values were expressed as mean ± SD. Statistical analyses for in vitro and in vivo experiments were performed using two-tailed Student’s t-test. The *p* values < 0.05 were considered significant.

## Supplementary Information

Supplementary Material 1: Supplementary Figure 1. Genomic editing by CRISPR/Cas9 in BRAF^V600^-mutant Mel 593 cell line. A, Spry1 expression was evaluated by Western blot analysis in Mel 593 parental and respective Spry1^KO^ clones. β-tubulin was used as a loading control. B, Western blot analysis of phospho-ERK1/2 (pERK1/2), ERK1/2, phospho-p38 (pp38), p38, bcl-2, and CCND1 in Mel 593 parental and respective Spry1^KO^ clones. β-Tubulin was used as a loading control.

Supplementary Material 2: Supplementary Figure 2. Induction of oxidative stress in BRAF^V600E^-mutant Mel 593 cells following Spry1^KO^. ROS levels were examined using flow cytometry in parental Mel 593 CM cell lines and respective Spry1^KO^ clones. Significance was evaluated with Student *t* test. **, *P* ≤ 0.01.

Supplementary Material 3: Supplementary Figure 3. HIF1α binding to Mel 593 cells chromatin. A, Heatmap showing the read density around the 10-kb regions centered on each HIF1α binding sites in Mel 593 cells, with respect to the control. B, Principal Component Analysis representing the three biological replicates of the IP HIF1α samples and of IP IgG. C, Histogram showing the distribution along the genome of HIF1α binding sites. D, PDK1 and LDHA protein levels from Fig. 3H presented normalized to b-tubulin. For the quantification analysis, the sum of the density of bands under study was calculated, and normalized to the amount of b-tubulin. After normalization with b-tubulin, changes in protein expression in Spry1^KO^ clones were calculated relative to the parental basal level. Significance was evaluated with Student *t* test. *, *P* ≤ 0.05. **, *P* ≤ 0.01.

Supplementary Material 4: Supplementary Figure 4. Spry1^KO^ effects in BRAF-mutant CM *in vivo*. A, Representative examples of tumors formed in nude mice following injection of Mel 593 parental (A, B) and Spry1^KO^ clone 1 (C) and clone 2 (D). B, Protein levels of PDK1 and LDHA from Fig. 5F, and of Keap1 from Fig. 5G presented normalized to b-tubulin. For the quantification analysis, the sum of the density of bands under study was calculated, and normalized to the amount of b-tubulin. After normalization with b-tubulin, changes in protein expression in Spry1^KO^ clones were calculated relative to the parental basal level. Significance was evaluated with Student *t* test. *, *P* ≤ 0.05. **, *P* ≤ 0.01.

Supplementary Material 5: Supplementary Figure 5. Macroscopic vascularization of BRAF-mutant CM *in vivo*. Mel 272 and Mel 611 parental and Spry1^KO^ representative tumors.

Supplementary Material 6.

Supplementary Material 7.

Supplementary Material 8.

Supplementary Material 9.

Supplementary Material 10.

Supplementary Material 11.

## Data Availability

The mass spectrometry proteomics data have been deposited to the ProteomeXchange Consortium via the PRIDE [76] partner repository with the dataset identifier PXD038916. All other raw data generated in this study are available upon request from the corresponding author.

## Abbreviations

ACK1: Activated CDC42 kinase 1
ALDOC: Aldolase C
BNIP3: BCL2 Interacting Protein 3
CM: Cutaneous melanoma
ECAR: Extracellular acidification rate
GLUT1: Glucose transporter protein type 1
HIF1α: Hypoxia-inducible factor-1 alpha
HK2: Hexokinase II
HUVEC: Human Umbilical Vein Endothelial Cells
IP: Immunoprecipitation
IPA: Ingenuity Pathway Analysis
LC–MS/MS: Liquid chromatography-tandem mass spectrometry
LDHA: Lactate dehydrogenase A
MMP: Matrix metalloproteinases
OCR: Oxygen consumption rate
OXPHOS: Oxidative phosphorylation
PDK1: Pyruvate dehydrogenase kinase 1
PGAM5: PGAM family member 5, mitochondrial serine/threonine protein phosphatase
PGK1: Phosphoglycerate kinase 1
ROS: Reactive oxygen species
siRNA: Small interfering RNA
Spry1^KO^: Spry1 knockout
TEM: Transmission electron microscopy
TME: Tumor microenvironment
VEGFA: Vascular endothelial growth factor A
wt: Wild-type
