# Correction: Suppression of Spry1 reduces HIF1α‑dependent glycolysis and impairs angiogenesis in BRAF‑mutant cutaneous melanoma

**DOI:** 10.1186/s13046-026-03800-9

**Published:** 2026-08-21

**Authors:** Barbara Montico, Giorgio Giurato, Roberto Guerrieri, Francesca Colizzi, Annamaria Salvati, Giovanni Nassa, Jessica Lamberti, Domenico Memoli, Patrizia Sabatelli, Marina Comelli, Arianna Bellazzo, Albina Fejza, Lucrezia Camicia, Lorena Baboci, Michele Dal Bo, Alessia Covre, Tuula A. Nyman, Alessandro Weisz, Agostino Steffan, Michele Maio, Luca Sigalotti, Maurizio Mongiat, Eva Andreuzzi, Elisabetta Fratta

**Affiliations:** 1 Immunopathology and Cancer Biomarkers Unit, Centro di Riferimento Oncologico di Aviano (CRO), IRCCS, Aviano, Italy; 2 Department of Medicine, Surgery and Dentistry ‘Scuola Medica Salernitana’, Laboratory of Molecular Medicine and Genomics, University of Salerno, Baronissi, Italy; 3 Genome Research Center for Health - CRGS, Baronissi, SA 84081 Italy; 4 Division of Oncology, AOU ‘S. Giovanni Di Dio E Ruggi 14 d’Aragona’, Universita Di Salerno, Molecular Pathology and Medical Genomics Program, Salerno, 84131 Italy; 5 CNR-Institute of Molecular Genetics “Luigi Luca Cavalli-Sforza”, Unit of Bologna, Bologna, Italy; 6 Department of Medicine, University of Udine, Udine, Italy; 7 Molecular Oncology Unit, Centro Di Riferimento Oncologico Di Aviano (CRO), IRCCS, Aviano, Italy; 8 UBT-Higher Education Institution, Street Rexhep Krasniqi Nr. 56, Prishtina, Kalabria 10000 Kosovo; 9 Experimental and Clinical Pharmacology Unit, Centro Di Riferimento Oncologico Di Aviano (CRO), IRCCS, Aviano, PN Italy; 10 University of Siena, Siena, Italy; 11 Department of Immunology, University of Oslo and Oslo University Hospital, Oslo, Norway; 12 Center for Immuno‑Oncology, University Hospital of Siena, Siena, Italy; 13 Oncogenetics and Functional Oncogenomics Unit, Centro Di Riferimento Oncologico Di Aviano (CRO), IRCCS, Aviano, Italy; 14 Obstetrics and Gynecology, Institute for Maternal and Child Health - IRCCS “Burlo Garofolo”, Trieste, 34137 Italy

**Correction: J Exp Clin Cancer Res 43, 303 (2024)**

**https://doi.org/10.1186/s13046-025-03289-8**

Following publication of the original article [1], the authors found errors in the published version of Fig. 1, specifically:

Fig. 1H – incorrect image was inadvertently uploaded. For Mel 272, the label “cytosolic proteins, n = 4” has been corrected to “n = 24”, while for Mel 593 “cytosolic proteins, n = 16” has been corrected to “n = 31”

The correct figure is presented below. The original article [1] has been corrected.

**Incorrect Fig. 1**

**Fig. 1:**
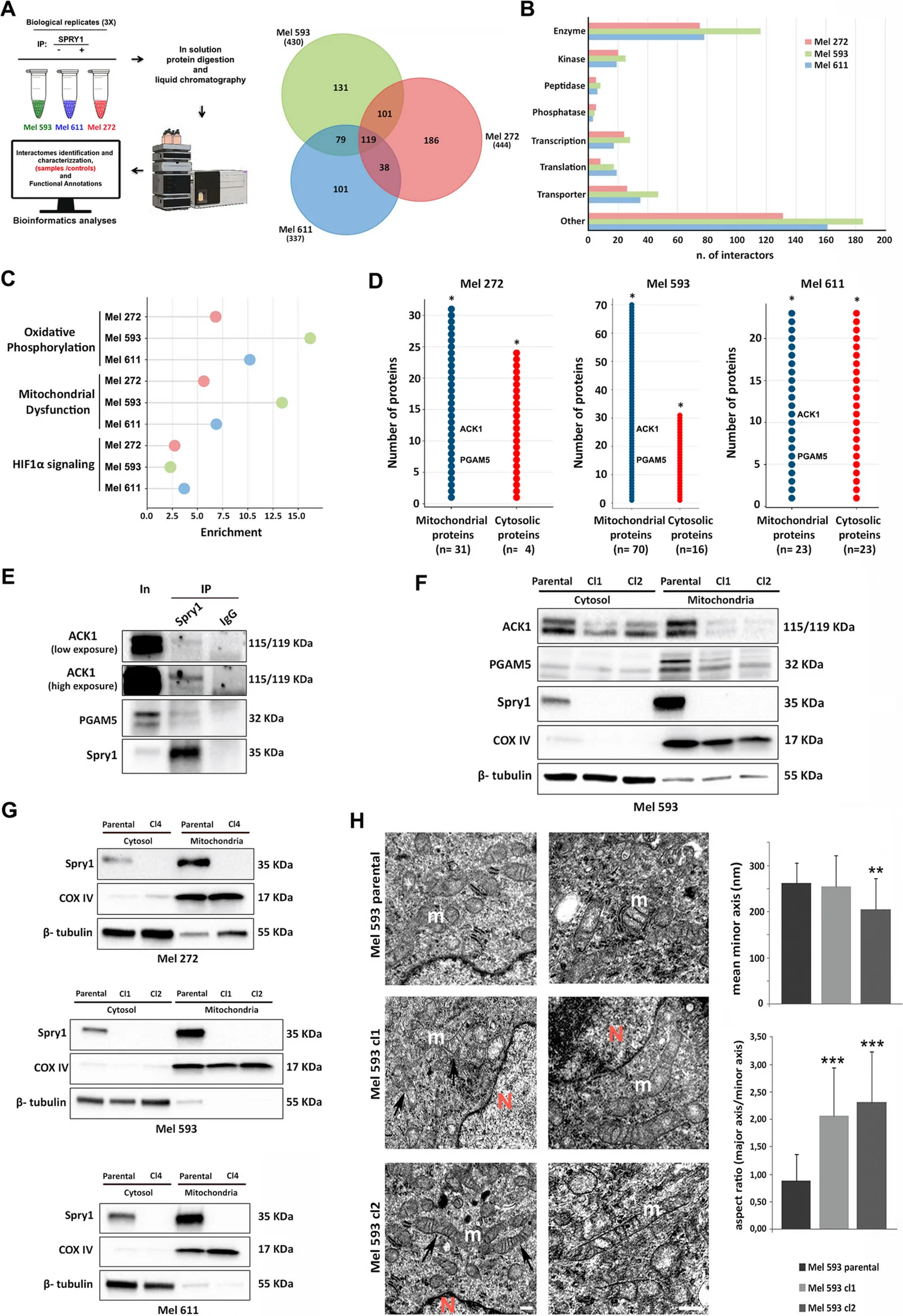
Spry1^KO^ is found associated to proteins involved in key mitochondrial functions in BRAF-mutant CM. **A** Summary of the experimental workflow applied to generate the protein datasets and venn diagram representing the Spry1-interacting proteins found enriched in Mel 272, Mel 593, and Mel 611 cells. **B** Classification of Spry1 molecular partners. **C** Lollipop chart showing statistically significant canonical pathways where the Spry1 interactomes are involved. The length of the lollipop indicates the enrichment value, the dot size corresponds to the number of proteins in each pathway. **D** Graph showing mitochondrial and cytosolic number of proteins in each of the Spry1 interactomes analysed; (* *P* ≤ 0.01, hypergeometric test). **E** IP-Western blot showing Spry1-ACK1 and Spry1-PGAM5 interaction. IgG was used as negative control. **F** Western blot analysis of ACK1, PGAM5 and Spry1 protein levels in cytosol and mitochondria in Mel 593 parental cells and Spry1^KO^ clones. COX IV and β-tubulin were used as protein loading control and as mitochondrial and cytosol markers, respectively. **G** Western blot analysis of Spry1 protein levels in cytosol and mitochondria in Mel 272, Mel 593, Mel 611 parental cells and Spry1^KO^ clones. COX IV and β-tubulin were used as protein loading control and as mitochondrial and cytosol markers, respectively. **H** TEM images of Mel 593 parental cells and their relative Spry1^KO^ clones. The arrows indicate m, mitochondrion; N, nucleus

**Correct Fig. 1**

**Fig. 1:**
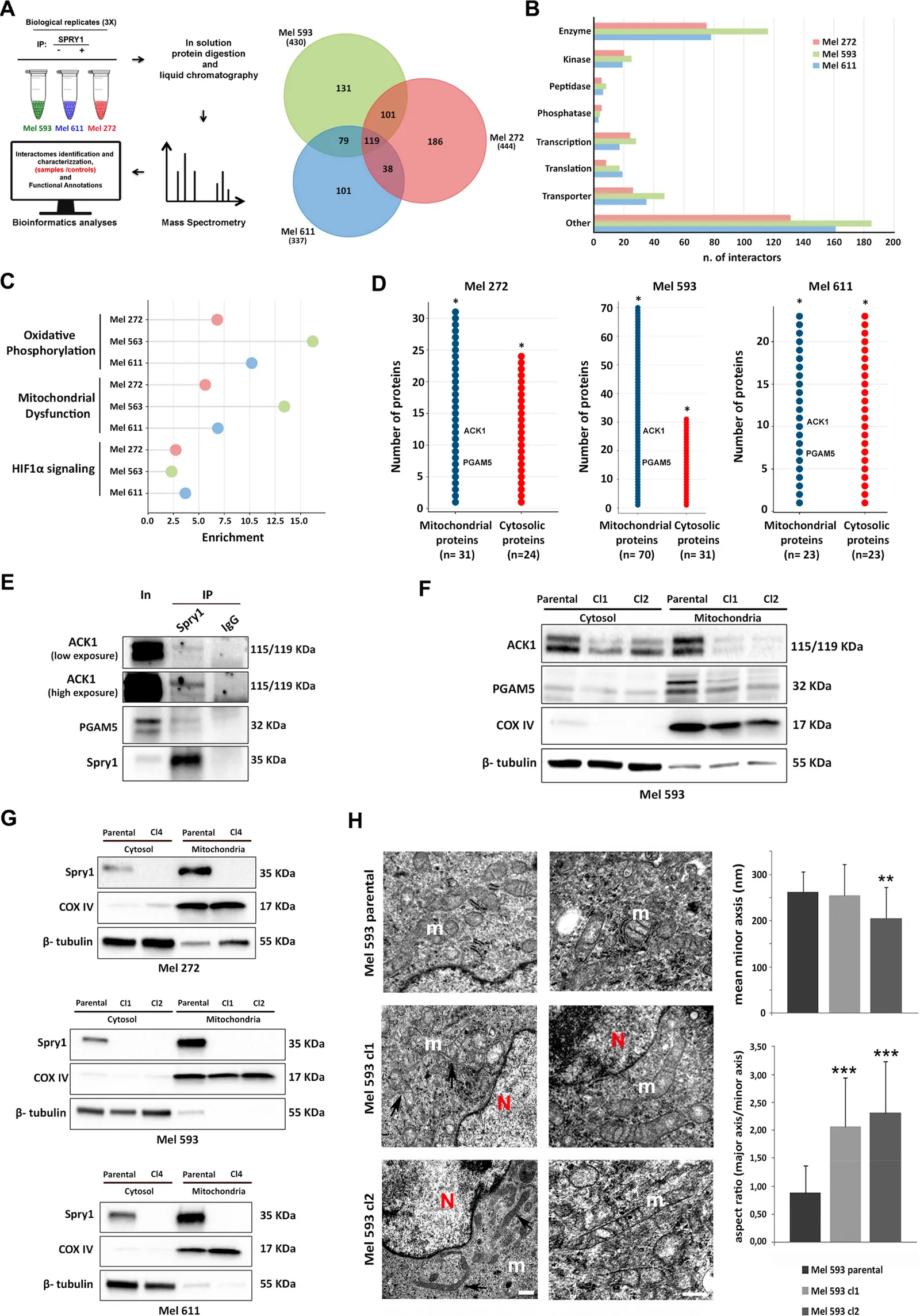
Spry1^KO^ is found associated to proteins involved in key mitochondrial functions in BRAF-mutant CM. **A** Summary of the experimental workflow applied to generate the protein datasets and venn diagram representing the Spry1-interacting proteins found enriched in Mel 272, Mel 593, and Mel 611 cells. **B** Classification of Spry1 molecular partners. **C** Lollipop chart showing statistically significant canonical pathways where the Spry1 interactomes are involved. The length of the lollipop indicates the enrichment value, the dot size corresponds to the number of proteins in each pathway. **D** Graph showing mitochondrial and cytosolic number of proteins in each of the Spry1 interactomes analysed; (* *P* ≤ 0.01, hypergeometric test). **E** IP-Western blot showing Spry1-ACK1 and Spry1-PGAM5 interaction. IgG was used as negative control. **F** Western blot analysis of ACK1, PGAM5 and Spry1 protein levels in cytosol and mitochondria in Mel 593 parental cells and Spry1^KO^ clones. COX IV and β-tubulin were used as protein loading control and as mitochondrial and cytosol markers, respectively. **G** Western blot analysis of Spry1 protein levels in cytosol and mitochondria in Mel 272, Mel 593, Mel 611 parental cells and Spry1^KO^ clones. COX IV and β-tubulin were used as protein loading control and as mitochondrial and cytosol markers, respectively. **H** TEM images of Mel 593 parental cells and their relative Spry1^KO^ clones. The arrows indicate m, mitochondrion; N, nucleus

## References

[1] Montico B, Giurato G, Guerrieri R, et al. Suppression of Spry1 reduces HIF1α-dependent glycolysis and impairs angiogenesis in BRAF-mutant cutaneous melanoma. J Exp Clin Cancer Res. 2025;44:53. 10.1186/s13046-025-03289-8. 39953610 10.1186/s13046-025-03289-8 PMC11827140

