# Supplementary figures and images for: Suppression of Spry1 reduces HIF1α-dependent glycolysis and impairs angiogenesis in BRAF-mutant cutaneous melanoma
Source: J Exp Clin Cancer Res. 2025 Feb 14;44:53. doi: 10.1186/s13046-025-03289-8 (PMC11827140; doi:10.1186/s13046-025-03289-8)

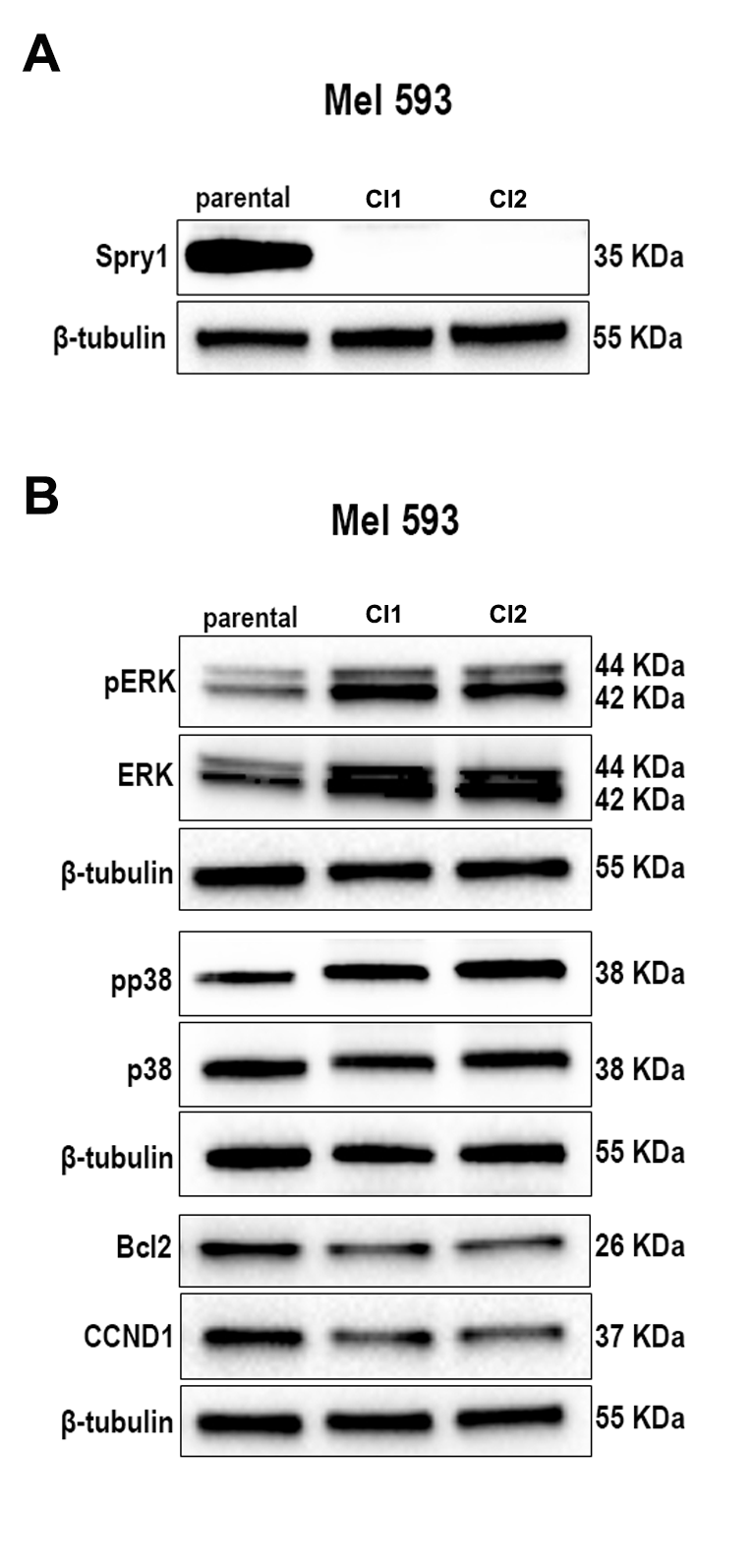

Supplement: Supplementary file 1 — Supplementary Material 1: Supplementary Figure 1. Genomic editing by CRISPR/Cas9 in BRAFV600-mutant Mel 593 cell line. A, Spry1 expression was evaluated by Western blot analysis in Mel 593 parental and respective Spry1KO clones. β-tubulin was used as a loading control. B, Western blot analysis of phospho-ERK1/2 (pERK1/2), ERK1/2, phospho-p38 (pp38), p38, bcl-2, and CCND1 in Mel 593 parental and respective Spry1KO clones. β-Tubulin was used as a loading control. [file 13046_2025_3289_MOESM1_ESM.png]

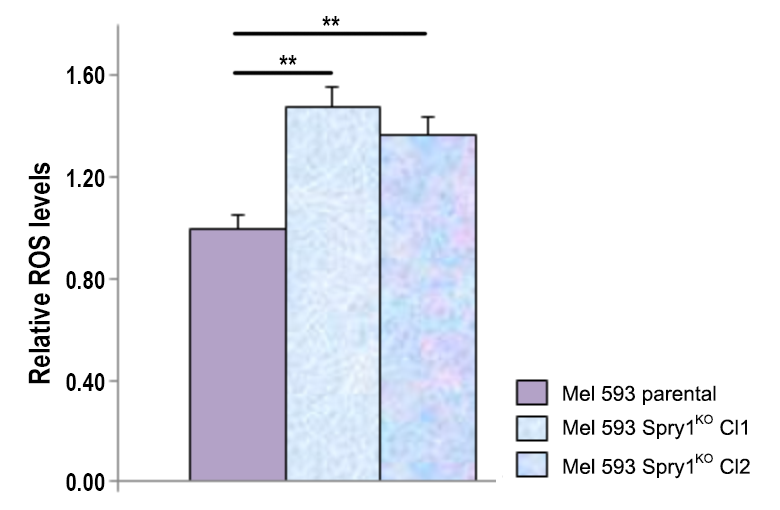

Supplement: Supplementary file 2 — Supplementary Material 2: Supplementary Figure 2. Induction of oxidative stress in BRAFV600E-mutant Mel 593 cells following Spry1KO. ROS levels were examined using flow cytometry in parental Mel 593 CM cell lines and respective Spry1KO clones. Significance was evaluated with Student t test. **, P ≤ 0.01. [file 13046_2025_3289_MOESM2_ESM.png]

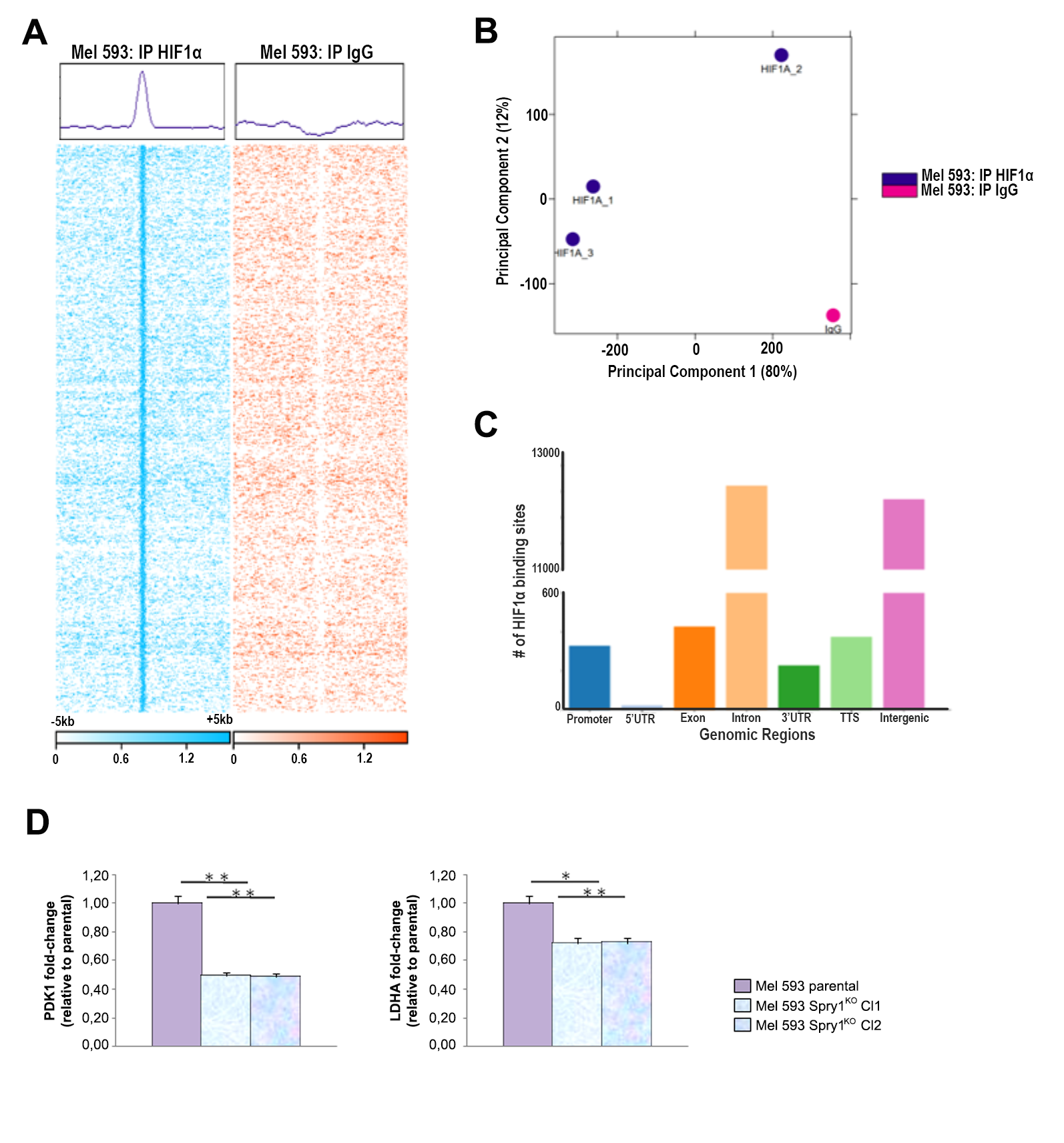

Supplement: Supplementary file 3 — Supplementary Material 3: Supplementary Figure 3. HIF1α binding to Mel 593 cells chromatin. A, Heatmap showing the read density around the 10-kb regions centered on each HIF1α binding sites in Mel 593 cells, with respect to the control. B, Principal Component Analysis representing the three biological replicates of the IP HIF1α samples and of IP IgG. C, Histogram showing the distribution along the genome of HIF1α binding sites. D, PDK1 and LDHA protein levels from Fig. 3H presented normalized to b-tubulin. For the quantification analysis, the sum of the density of bands under study was calculated, and normalized to the amount of b-tubulin. After normalization with b-tubulin, changes in protein expression in Spry1KO clones were calculated relative to the parental basal level. Significance was evaluated with Student t test. *, P ≤ 0.05. **, P ≤ 0.01. [file 13046_2025_3289_MOESM3_ESM.png]

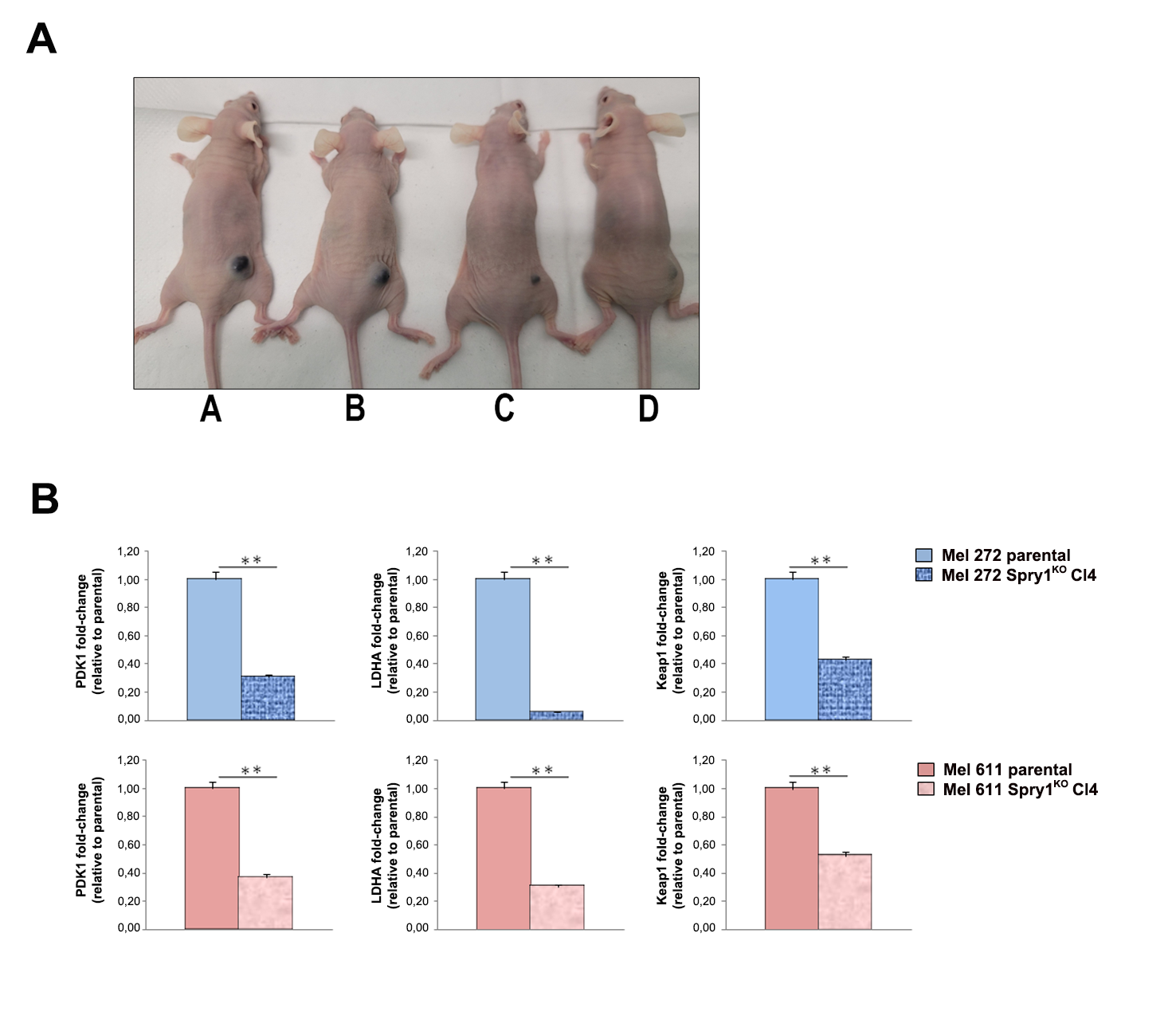

Supplement: Supplementary file 4 — Supplementary Material 4: Supplementary Figure 4. Spry1KO effects in BRAF-mutant CM in vivo. A, Representative examples of tumors formed in nude mice following injection of Mel 593 parental (A, B) and Spry1KO clone 1 (C) and clone 2 (D). B, Protein levels of PDK1 and LDHA from Fig. 5F, and of Keap1 from Fig. 5G presented normalized to b-tubulin. For the quantification analysis, the sum of the density of bands under study was calculated, and normalized to the amount of b-tubulin. After normalization with b-tubulin, changes in protein expression in Spry1KO clones were calculated relative to the parental basal level. Significance was evaluated with Student t test. *, P ≤ 0.05. **, P ≤ 0.01. [file 13046_2025_3289_MOESM4_ESM.png]

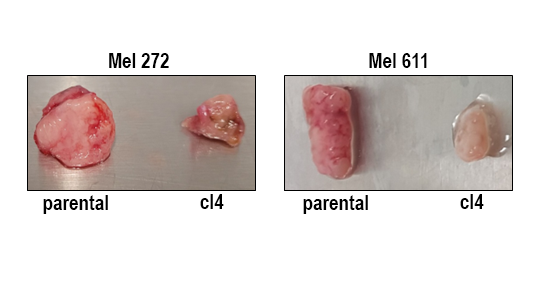

Supplement: Supplementary file 5 — Supplementary Material 5: Supplementary Figure 5. Macroscopic vascularization of BRAF-mutant CM in vivo. Mel 272 and Mel 611 parental and Spry1KO representative tumors. [file 13046_2025_3289_MOESM5_ESM.png]
