# Supplementary material for: Suppression of Spry1 reduces HIF1α-dependent glycolysis and impairs angiogenesis in BRAF-mutant cutaneous melanoma
Source: J Exp Clin Cancer Res. 2025 Feb 14;44:53. doi: 10.1186/s13046-025-03289-8 (PMC11827140; doi:10.1186/s13046-025-03289-8)
Supplement: Supplementary file 10 — Supplementary Material 10. [file 13046_2025_3289_MOESM10_ESM.docx]

Supplementary Table S5. Primers used for qPCR and PCR analyses

| **Gene** | **Forward** | **Reverse** |
| --- | --- | --- |
| qPCR | | |
| β-actin | CGAGCGCGGCTACAGCTT | CCTTAATGTCACGCACGATT |
| ALDOC | ATGCCTCACTCGTACCCAG | TTTCCACCCCAATTTGGCTCA |
| BNIP3 | CAGGGCTCCTGGGTAGAACT | CTACTCCGTCCAGACTCATGC |
| GLUT1 | TTGCAGGCTTCTCCAACTGGAC | CAGAACCAGGAGCACAGTGAAG |
| HK2 | GAGTTTGACCTGGATGTGGTTGC | CCTCCATGTAGCAGGCATTGCT |
| LDHA | GTTGGTGCTGTTGGCATGGC | GTGATAATGACCAGCTTGGAG |
| PDK1 | ACCACGAGGCTGATGACTG | GGAACGTCGTCATGTCTTTG |
| PGK1 | TGCAAAGGCCTTGGAGAG | TGGATCTTGTCTGCAACTTTAGC |
| HIF1α | GTTAGTTCAATTTTGATCCCCTTTCT | GCTACTGCAATGCAATGGTTTAA |
| MMP2 | CCCCAAAACGGACAAAGAG | CTTCAGCACAAACAGGTTGC |
| MMP3 | GAAATGAGGTACGAGCTGGATACC | ATGGCTGCATCGATTTTCCT |
| MMP8 | CAACCTACTGGACCAAGCACAC | TGTAGCTGAGGATGCCTTCTCC |
| SPRY1 | CCTCCTGAACTTTTAGCTTTCAA | GGAAAAATTCTAAAGAAAACAAAAACA |
| VEGFα | CTACCTCCACCATGCCAAGT | GCAGTAGCTGCGCTGATAGA |
| PCR | | |
| PDK1 | AGGCTGACCCTGTATCTGGT | AAGTTCCCCTATCCCCTGCT |
