# Supplementary material for: Suppression of Spry1 reduces HIF1α-dependent glycolysis and impairs angiogenesis in BRAF-mutant cutaneous melanoma
Source: J Exp Clin Cancer Res. 2025 Feb 14;44:53. doi: 10.1186/s13046-025-03289-8 (PMC11827140; doi:10.1186/s13046-025-03289-8)
Supplement: Supplementary file 11 — Supplementary Material 11. [file 13046_2025_3289_MOESM11_ESM.doc]

Supplementary Table S6. Antibodies used for Western Blot analyses

| **Antibody name** | **Vendor** | **Catalogue number** |
| --- | --- | --- |
| Rabbit anti-phospho-p42/44 MAPK (Erk1/2) (Thr202/Tyr204) | Cell Signaling Technology | #9101 |
| Rabbit anti-p42/44 MAPK (Erk1/2) | Cell Signaling Technology | #9102 |
| Rabbit anti-Spry1 (D9V6P) | Cell Signaling Technology | #13013 |
| Rabbit anti-Histone H1.4 (D4J5Q) | Cell Signaling Technology | #41328 |
| Rabbit anti-HIF-1α (D1S7W) | Cell Signaling Technology | #36169 |
| Rabbit anti-phospho-p38 MAPK (Thr180/Tyr182) | Cell Signaling Technology | #9211 |
| Rabbit anti-p38 MAPK (D13E1) | Cell Signaling Technology | #8690 |
| Rabbit anti-LDHA (C4B5) | Cell Signaling Technology | #3582 |
| Rabbit anti-KEAP1 (D6B12) | Cell Signaling Technology | #8047 |
| Mouse anti-β-Tubulin (D3U1W) | Cell Signaling Technology | #86298 |
| Mouse anti-Bcl2 (124) | Cell Signaling Technology | #15071 |
| Rabbit anti-Cyclin D1 (92G2) | Cell Signaling Technology | #2978 |
| Rabbit anti-Cox IV (3E11) | Cell Signaling Technology | #4850 |
| Mouse anti-ACK (A-11) | Santa Cruz Biotechnology | sc-28336 |
| Mouse anti-PGAM5 (A-3) | Santa Cruz Biotechnology | sc-515880 |
| Rabbit anti-PDK1/PDHK1 | ABclonal | A0834 |
